# Quaternary E‐W Extension Uplifts Kythira Island and Segments the Hellenic Arc

**DOI:** 10.1029/2022TC007231

**Published:** 2022-10-11

**Authors:** Gino de Gelder, David Fernández‐Blanco, Nazik Öğretmen, Spyros Liakopoulos, Dimitris Papanastassiou, Costanza Faranda, Rolando Armijo, Robin Lacassin

**Affiliations:** ^1^ ISTerre Université Grenoble‐Alpes St. Martin d’Hères; ^2^ Université Paris cité Institut de physique du globe de Paris CNRS Paris France; ^3^ Research Group of Paleoclimate & Paleoenvironment Research Centre for Climate and Atmosphere, Research Organization of Earth Sciences and Maritime, National Research and Innovation Agency (BRIN) Bandung Indonesia; ^4^ Barcelona Center for Subsurface Imaging Passeig Marítim de Barceloneta 37‐49 Barcelona Spain; ^5^ Eurasia Institute of Earth Sciences Istanbul Technical University Istanbul Turkey; ^6^ Dipartimento di Scienze Università degli Studi Roma Tre Rome Italy; ^7^ Institute of Geodynamics National Observatory of Athens Athens Greece

**Keywords:** Kythira, Hellenic Arc, marine terraces, sedimentary basins, uplift, normal faults

## Abstract

Several crustal and lithospheric mechanisms lead to deformation and vertical motion of the upper plate during subduction, but their relative contribution is often enigmatic. Multiple areas of the Hellenic Forearc have been uplifting since Plio‐Quaternary times, yet spatiotemporal characteristics and sources of this uplift are poorly resolved. The remarkable geology and geomorphology of Kythira Island, in the southwestern Hellenic forearc, allow for a detailed tectonic reconstruction since the Late Miocene. We present a morphotectonic map of the island, together with new biostratigraphic dating and detailed analyses of active fault strikes and marine terraces. We find that the Tortonian‐Pliocene stratigraphy in Kythira records ∼100 m of subsidence, and a wide coastal rasa marks the ∼2.8–2.4 Ma maximum transgression. Subsequent marine regression of ∼300–400 m and minor E‐W tilt are recorded in ∼12 marine terrace levels for which we estimate uplift rates of ∼0.2–0.4 mm/yr. Guided by simple landscape evolution models, we interpret the coastal morphology as the result of initial stability or of slow, gradual sea‐level drop since ∼2.8–2.4 Ma, followed by faster uplift since ∼1.5–0.7 Ma. Our findings on‐ and offshore suggest that E‐W extension is the dominant mode of regional active upper crustal deformation, and N‐S normal faults accommodate most, if not all of the uplift on Kythira. We interpret the initiation of E‐W extension as the result of a change in plate boundary conditions, in response to either propagation of the North Anatolian Fault, incipient collision with the African plate, mantle dynamics or a combination thereof.

## Key Points

- ∼100 m of Tortonian‐Pliocene subsidence of Kythira Island was followed by ∼300–400 m of uplift since ∼2.6 Ma
- We propose that regional E‐W extension, caused by large‐scale tectonic changes ∼1.5–0.7 Ma, is the dominant driver to uplift Kythira Island
- We highlight the general importance of upper crustal faulting as a major process of forearc uplift

## 1 Introduction

The mode of deformation of the upper plate during subduction and the mechanisms of uplift at the front of the overriding plates are two fundamental questions that remain unresolved (e.g., Davis et al., 1983; Fuller et al., 2006; Gallen et al., 2014; Gutscher et al., 1996; Larroque et al., 1995; Willet et al., 1993). Forearc uplift may be driven by upper crustal faults (e.g., Armijo et al., 2015), sedimentary underplating (e.g., Menant et al., 2020), lower crustal mantle flow (e.g., Fernández‐Blanco et al., 2020), slab dynamics (e.g., Guillaume et al., 2013) and/or dynamic topography (e.g., Conrad & Husson, 2009), and each of these have particular implications in terms of seismicity and for structure and mountain building in general. The Hellenic subduction has the largest convergence velocity and holds the highest seismic activity among Mediterranean arcs (McClusky et al., 2000; Reilinger et al., 2006), and thus, its forearc is key in this debate. The steep normal faults that control the present‐day structure and seismicity of the Hellenic Arc are oblique to the intraplate contact in the SW of the arc, transecting former fold‐and‐thrust structures that are parallel to the trench, but are parallel or perpendicular to the arc in other sections (Armijo et al., 1992; de Chabalier et al., 1992; Lyon‐Caen et al., 1988; Shaw & Jackson, 2010). Constraining the timing and kinematics of these normal faults, and of the recent structural evolution of the arc between uplifted islands and offshore grabens, is key to better understand the mechanics of the Hellenic plate boundary. Important regional tectonic processes that have been put forward include slab rollback (e.g., Angelier et al., 1982), sedimentary underplating (e.g., Gallen et al., 2014), slab tearing in the W‐Hellenic Arc (e.g., Jolivet et al., 2013), incipient collision with the African Plate (e.g., Lyon‐Caen et al., 1988) and the propagation of the North Anatolian Fault (e.g., Flerit et al., 2004).

**Figure 1:**
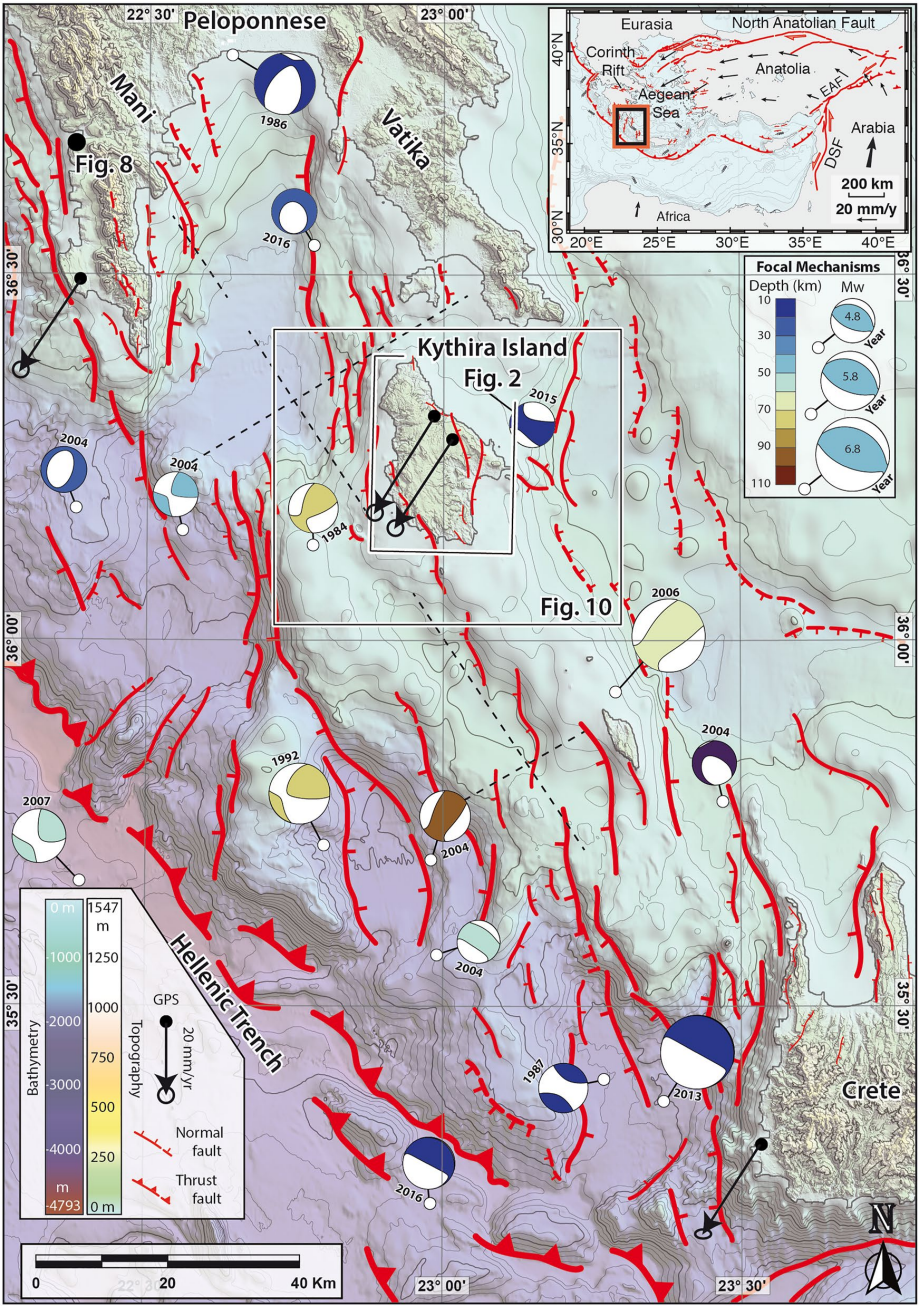
Active tectonics of the SW Hellenic Arc. Topography is an ALOS Global Digital Surface Model (DSM), and bathymetry is from the European Marine Observation and Data Network (EMODNet) Digital Terrain Model (DTM). Offshore fault mapping is an interpretation of the bathymetry based on relief offset and morphology, and accounts for fault mapping by Lyberis et al. (1982) and Armijo et al. (1992). Focal mechanisms (>4.5 Mw since 1976, colored by depth and sized by magnitude) are build using both the main and the monthly curated databases of the Global Centroid Moment Tensor (GCMT) catalog (https://www.globalcmt.org/; Ekström et al., 2012), as downloaded on the 14 December 2020. GPS vectors are relative to stable Eurasia, and taken from England et al. (2016), but we note that these would be similar in direction and magnitude if re‐calculated with respect to stable Nubia (Reilinger et al., 2006; Shaw & Jackson, 2010). Dashed lines are the seismic lines of Kokinou and Kamberis (2009). Although not important to our study, note that we interpret the Hellenic Trench as a thrust fault system (as in Shaw et al., 2008; Mouslopoulou et al., 2015), but others have considered it as an extensional or transtensional structure (e.g., Gallen et al., 2014; Lallemant et al., 1994) with the surface expression of the Hellenic subduction zone ∼200 km further S. The location of Figures 2, 8 and 10 is indicated.

Kythira is the largest island between SW Peloponnese and NE Crete (Figure 1), and provides an exceptional but surprisingly unattended opportunity to understand the interaction between past and active tectonics in the Hellenic Arc. Kythira's sedimentary record and morphological characteristics allow a detailed reconstruction of its tectonic evolution since the Late Miocene. Whereas emerged marine sedimentary basins record the island's vertical motions during the Neogene (Van Hinsbergen et al., 2006), its landscape archives a recent uplift phase evidenced by coastline abrasion surfaces, marine terraces and steep river gorges. Holocene fault scarps and numerous historical earthquakes evidence active faulting (Papadopoulos & Vassilopoulou, 2001; Veliz‐Borel et al., 2022), but controversy remains on the processes that drove the initiation of the main fault systems, both on the island itself and within the regional context of the Hellenic Arc. Current propositions for dominant modes of normal faulting include arc‐parallel faulting (e.g., Veliz‐Borel et al., 2022), arc‐parallel combined with arc‐perpendicular faulting (e.g., Marsellos & Kidd, 2008) and N‐S oriented faulting (e.g., Armijo et al., 1992). Previous assessment of the vertical motion history of the island suggests a period of rapid Early Pleistocene uplift followed by a period of slow uplift (Van Hinsbergen et al., 2006). No combined sedimentary and geomorphic investigation has previously been carried out to present an integrated perspective of Kythira's faults, sedimentary basins and marine terraces.

We reconstruct the Late Miocene‐Recent tectonic evolution of Kythira through a multi‐disciplinary approach that includes morphometric and basin analyses, fieldwork, biostratigraphic dating and numerical modeling. We developed a 2 m‐resolution Digital Surface Model (DSM) from Pleiades satellite imagery that allows us to detect and map geologic and geomorphic features like faults, marine terraces, drainage systems and stratigraphic layering with unprecedented detail. We present a detailed geological, structural and geomorphological map of Kythira Island, and a comprehensive analysis of marine terraces based on a combination of fieldwork and the high resolution DSM. We constrain absolute ages and evaluate the rates of surface uplift dating marine deposits near the highest and most extensive marine abrasion surface of the island with microfossils. Combining these ages and the coastal morphology, we then use simple landscape evolution models to test different uplift scenarios. This allows us to put forward a detailed discussion on the tectonic changes that have affected the island, and discuss our findings in the context of the evolution of the Hellenic Arc.

## 2 Tectonic, Geologic, and Geomorphic Setting

Subduction of the African plate below the Aegean has stacked the Hellenic fold‐and‐thrust belt nappes since the early Mesozoic (e.g., Aubouin, 1957; Faccenna et al., 2003; Jacobshagen, 1987; Jolivet & Brun, 2010; van Hinsbergen et al., 2005). Three of those nappes are exposed on Kythira: the Arna Unit, the Tripolis Unit and the Pindos Unit (Danamos, 1992; Manolessos, 1955; Papanikolaou & Danamos, 1991). The HP/LT metamorphic Arna Unit (Gerolymatos, 1994; Marsellos et al., 2014; Marsellos & Kidd, 2008) is highly folded and crops out in the north of the island. Overthrusting the Arna Unit, the Tripolis Unit contains Jurassic‐Eocene limestones, dolomites and flysch (Danamos, 1992). The Tripolis Unit crops out along large parts of the NE and SW coasts and in the center of the island, typically along NW‐SE trending ridges parallel to the Hellenic trench. The Pindos Unit overthrusts both the underlying Arna and Tripolis units and contains Cretaceous‐Early Cenozoic limestones and flysch (Danamos, 1992). The Pindos Unit mainly crops out in the central and SE parts of the island, and has a NW‐SE trend similar to the Tripolis Unit. Apatite and zircon fission track cooling ages for the metamorphic Arna Unit indicate exhumation during the Middle‐Late Miocene (Marsellos et al., 2014; Marsellos & Kidd, 2008). The oldest preserved Neogene sediments on Kythira Island are of Tortonian age (Meulenkamp et al., 1977; Theodoropoulos, 1973; van Hinsbergen et al., 2006), and were probably deposited not long after exhumation ceased.

**Figure 2:**
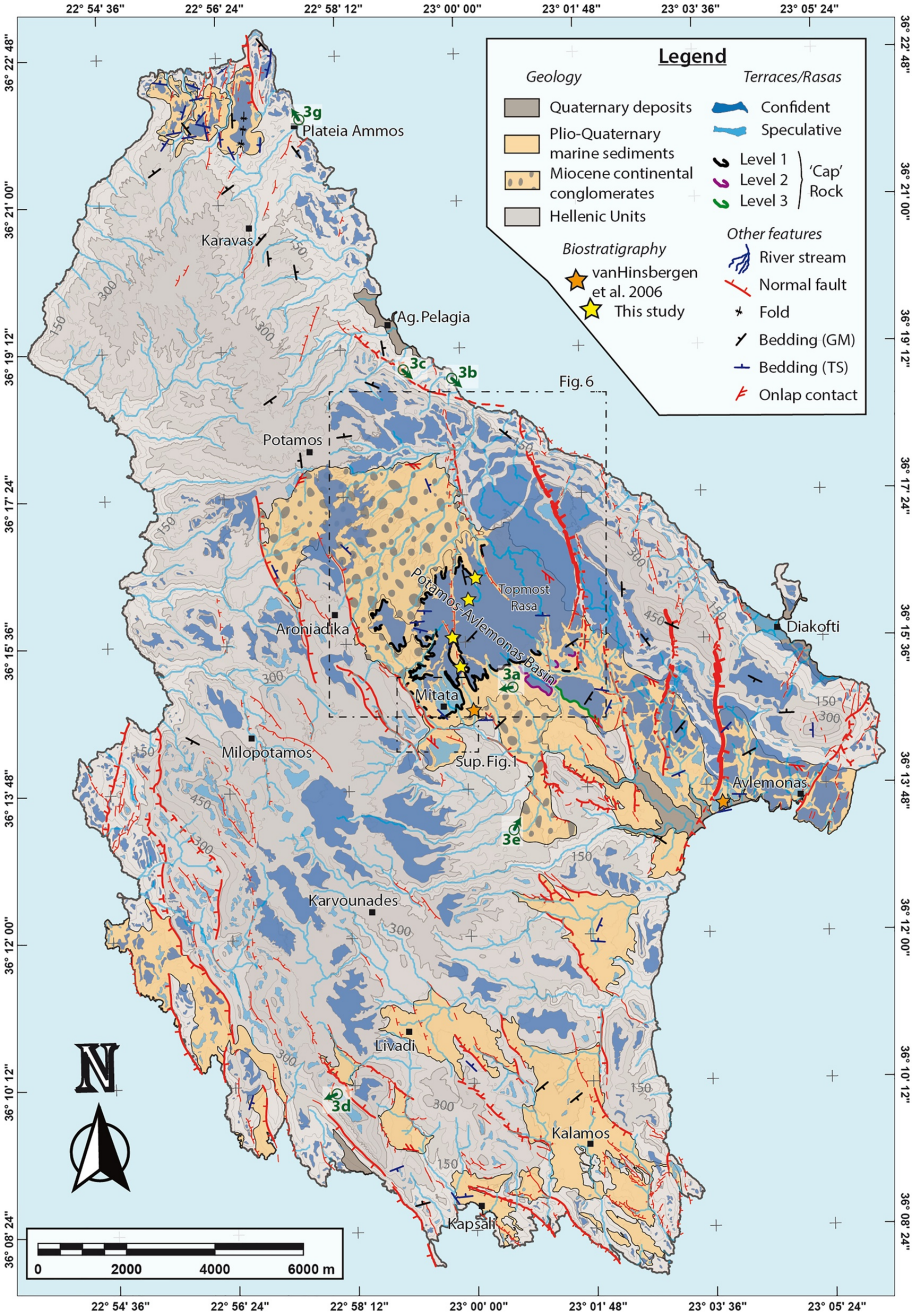
Active tectonics on Kythira. Map with main geologic and geomorphic features, based on 2m‐resolution Digital Surface Model developed from Pleiades satellite imagery and fieldwork (see text). Topographic contours are given at 50 m intervals, with color changes every 150 m. Active faults in red have different stroke thickness to show 3 levels of relative offset, and most present fault scarps as corroborated in situ during fieldwork. Marine terraces and the topmost rasa levels are shown in two tones of blue, with a lighter tone for more speculative surfaces (see main text). Bedding measurements are both from this study (TS) and the geological map (GM) of Danamos (1992). Observed onlap contacts between the largest uplited basin and basement are also shown. Insets show locations of Figure 6 and Figure S1 in Supporting Information S1. Green symbols mark the field photographs of Figure 3 with viewing direction marked with arrows. Yellow stars mark the locations of samples dated by biostratigraphy (see results on Figure 6); orange stars, near Mitata and W of Avlemonas, show location of Van Hinsbergen et al. (2006) sites.

Neogene‐Quaternary rocks unconformably overlie the Hellenic nappes and are scattered around the island, with the largest basin located in its central‐eastern sector, between Potamos and Avlemonas (Figure 2). The Neogene stratigraphy has previously been described as a Tortonian terrigenous‐clastic succession at the bottom overlaid in angular unconformity by a Pliocene calcareous succession (Meulenkamp et al., 1977; Theodoropoulos, 1973). Paleobathymetry estimates from a Plio‐Pleistocene calcareous section near the present‐day coast at Avlemonas (orange star on Figure 2) suggest deepening from ∼300 to ∼750 m depth between ∼3.5 and ∼2.5 Ma (van Hinsbergen et al., 2006).

The highest elevated sediments of the calcareous succession form part of a large marine rasa (Figure 2), i.e. a wide coastal planation surface of polygenic origin formed by sea erosion during multiple sea‐level stands (e.g., Dawson et al., 2013; Guilcher, 1974; Pedoja et al., 2006; Regard et al., 2010). The rasa in Kythira is carved into both basement nappes and Neogene sediments (Figure 2), is most extensive around the airport (NE of Mitata), and can be found all around the island between elevations of ∼200 and ∼400 m. It forms the highest marine abrasion surface in a flight of marine terraces that reaches the present‐day coastline, which is best preserved along the east coast. Gaki‐Papanastassiou et al. (2011) described 6–8 different terrace levels in the southern part of the island, with sea caves and notches at 0.4–0.6, 2, and 4 m above sea level evidencing recent uplift. They note that terraces are at a generally higher elevation along the W‐coast compared to the E‐coast, which they relate to the subduction zone SW of Kythira, and estimate a minimum Quaternary uplift rate of ∼0.13 mm/yr.

The active faults on the island have been described as NW‐SE trending normal faults related to trench‐normal extension, with a Miocene detachment fault indicating older trench‐parallel extension (Marsellos & Kidd, 2008), or NNW‐SSE trending normal faults related to N‐NE directed subduction (Gaki‐Papanastassiou et al., 2011). A recent paper by Veliz‐Borel et al. (2022) analysed the faults on Kythira in detail, and found three groups of active faults that all display almost pure dip‐slip motion: Group A consists of NW‐SE striking normal faults that are the longest on the island and determine the topography, Group B of N‐S striking normal faults that are shorter and abut against the Group A faults, and Group C of WNW‐ESE striking normal faults that are less common and also abut against Group A faults. They compare slip rate over Holocene and Quaternary timescales for 28 faults on the island, and find that slip rates for the two timescales differ over an order of magnitude for individual faults, but displacement accumulation integrated over the scale of the island is more constant.

Historical earthquakes in Kythira have been documented in 800 AD, 1750, 1798, 1866 and 1903 (Gaki‐Papanastassiou et al., 2011), but are difficult to associate with the subduction zone or specific upper crustal faults. The 2006 magnitude 6.7 Mw earthquake that caused significant destruction to the town of Mitata (Figure 2) had a focal depth of ∼60 km (Figure 1), and is thus probably unrelated to any of the faults observed on the island (Konstantinou et al., 2006). Most focal mechanisms from regional earthquakes of the past decades are difficult to link directly to faults traced at the surface (Figure 1), but a few earthquakes can be clearly related to N‐S striking normal faults (Figure 1; the 1986 and 2016 earthquakes between Vatika and Mani, and the 2004 earthquake NW of Crete).

## 3 Methods

### 3.1 Developing and Analysing High‐Resolution DSMs Derived From Pleiades Imagery

Tri‐stereo Pleiades satellite images of 0.5 m resolution covering the whole island of Kythira were obtained through the ISIS and Tosca programs of the Center National d’Etudes Spatiales (CNES, France). The open‐source software MicMac (Rosu et al., 2015; Rupnik et al., 2016) was used to create tie‐points, orientate the images and calculate 0.5 m‐resolution DSMs, using ground control points at 0 m elevation for several locations along the coastline. To reduce the topographic effects of vegetation, crops and man‐made structures, the DSMs were downsampled to 2 m resolution. Objects of ∼50 cm height are easily detected, indicating a relative vertical accuracy of less than 1 m. The accuracy and refinement of our geologic/geomorphic map is relevantly improved thanks to usage of the Pleiades DSM in comparison to freely available digital elevation models (Figure S1 in Supporting Information S1). In addition, we also used stacked swaths of various sectors of the Pleiades DSM to gain a “2.5D” view of the topography. Stacked swaths (Armijo et al., 2015; Fernández‐Blanco et al., 2019b) contain a significant number of parallel swath profiles derived from topographic data, which are plot together orthogonal to their strike as hairlines. By stacking swath profiles, the resulting profile highlights topographic coherence in depth (perpendicular to the viewpoint), allowing the distinction of structural and morphological features that are continuous over large scales. We share a georeferenced hillshade image and slope map of the 2 m‐resolution Digital Surface Model through these links: https://doi.org/10.6084/m9.figshare.18715535.v1 (hillshade image) and https://doi.org/10.6084/m9.figshare.18714914.v1 (slope map). The map of Figure 2 can be downloaded in georeferenced format (as Geospatial PDF) at https://doi.org/10.6084/m9.figshare.18703496.v1.

### 3.2 Active Faults and Sedimentary Basins

We carried out a detailed, albeit preliminary, mapping of faults, unit contacts and marine terraces and rasas by analyzing a combination of satellite imagery and DSM‐derived hillshade maps, slope maps and topographic profiles. We carefully mapped contacts between the basement nappes and sedimentary basins as well as contacts within the basins, refining them from previous maps (Geological Map of Kythira Island – IGME, 1966; Danamos, 1992). During this stage prior to fieldwork, we also mapped the terraces semi‐automatically, using the slope and roughness of the topography as guidelines, but evaluating every mapped terrace manually (Section 3.4). We identified the relative offset of faults using the topographic difference in parallel direction to the larger slope, corroborated with cross‐sections along the same direction on occasion.

Fieldwork was carried out to verify and improve the mapped structures, study the terrace and fault morphologies in detail, resolve the stratigraphic contacts of Neogene deposits with the basement rocks, and understand the overall tectonic architecture of the sedimentary basins. All preliminary mapped faults were checked in the field to corroborate its correct mapping, and most faults presented a (sometimes degraded) fault scarp. We focused our analysis of the stratigraphic succession on the largest basin between Potamos and Avlemonas. We took bedding measurements at representative locations and observed kinematic indicators on fault scarps to understand their sense of motion. We mapped the offshore faults based on a 120‐m resolution bathymetry dataset from the European Marine Observation and Data Network (EMODNet). The seismic sections that intersect with our mapped lineaments (Figure 1) evidence that these are active faults (Kokinou & Kamberis, 2009). Although we do not have direct evidence that the other mapped offshore lineaments are active faults, based on the similarity in laterally sustained topographic gradient, slope, size and orientation we also interpret the mapped lineaments without seismic images as active faults.

To quantify the overall strike of the active faults on Kythira, we subdivided them in 200‐m segments, roughly corresponding to the smallest mapped faults, and placed them in bins of 5°. We choose to have a larger statistical representation of larger, regional faults over smaller equivalents to gain a more accurate understanding on the dominant displacements as set by strike direction of their accomodating faults. Longer faults accommodate more displacement and are more likely to represent regional stresses, whereas the orientation of shorter faults is more likely to be influenced by local heterogeneities, damage zones around fault tips, shallow (<1 km) gravitational forces etc. The same was done for all faults mapped on‐ and off‐shore to the NE of the Hellenic Trench (Figure 2) with 4‐km long fault segments. Fault dip directions should be perpendicular to the strike of active normal faults. As a coarser but more objective alternative to measure the dominant fault strike offshore, we also quantified the slope direction of the bathymetry NE of the Hellenic Trench, calculating the 3x3 pixel slope direction for every ∼200 × 200 m pixel. We used the EMODNet bathymetry, and the slope direction is calculated in Global Mapper® by fitting a plane through each set of 3 x 3 pixels. To a first order, the slope direction of steep slopes should be perpendicular to the strike of active faults.

### 3.3 Biostratigraphic Dating

Marine foraminifer and ostracod species typically live(d) within a restricted water depth range, and within restricted time‐intervals throughout geological history. As such, their occurrence in marine sediments ‐ if not re‐worked ‐ can provide constraints on the age and/or paleodepth of those sediments. We sampled the deposits immediately below the uppermost marine sediments at 5 different sites for microfossil analysis (planktic and benthic foraminifers and ostracods), to obtain an approximate age for the formation of the rasa in locations around the area NE of Mitata (yellow stars on Figure 2). These uppermost sediments typically form a 2‐to‐5 m‐thick layer of coarse marine sandstones, rich in shells and oysters, acting as a caprock to the underlying finer sandstones that were sampled (Figure 2).

Samples were soaked in a H_2_O_2_ 5% solution for 24–48 hr, sieved under tap water using 0.066 and 0.125 mm mesh sieves, and dried in a 40°C oven. Whenever possible, up to 300 individuals were hand‐picked from the dry sieved samples under a stereomicroscope, in order to collect a significant sample of the thanatocoenosis. The taxonomic identification of planktic foraminifers was based on Parker (1962), Postuma (1971), Kennett and Srinivasan (1983), Iaccarino et al. (2007), and Lirer et al. (2019). Benthic foraminifer classification was based on AGIP (1982), Morkhoven et al. (1986), and Meriç et al. (2014). For further visual comparison, we used the foraminifer databases of WoRMS, Foraminifera.eu, and www.microtax.org. The biostratigraphy of the study area was updated following the planktic foraminifer biozones of Lirer et al. (2019) and the Global Time Scale 2012 edited by Anthonissen and Ogg (2012). We took several pictures for identification under the Scanning Electron Microscope Philips XL30 (Figure S2 in Supporting Information S1).

### 3.4 Marine Terrace Analysis

Marine terraces are geomorphic markers that record the former position of the sea‐level, and can thus be used to derive relevant information about vertical tectonic movements (e.g., Armijo et al., 1996; de Gelder et al., 2019; Dupré, 1984). The marine terraces on Kythira are wave‐cut terraces, which are typically formed by wave‐abrasion during sea‐level rise and highstands (Anderson et al., 1999) and expressed as smooth planar surfaces with slope angles of 1°–15° (Scott & Pinter, 2003) that may be covered by a thin layer of sediments.

To map marine terraces, we used the surface classification model (SCM) of Bowles and Cowgill (2012) as a guideline to select surfaces with a low slope and roughness. After smoothing the DSM with a 6 × 6 m moving window, a roughness threshold of 3.5 was used to incorporate 90% of the data, and a slope threshold of 6° was used to avoid misinterpreting degraded paleo‐cliffs, locally dipping as little as ∼7°–9°. After removing the SCM surfaces that were clearly not terraces (e.g., man‐made surfaces), high‐confidence terraces were separated from more speculative terraces (strong and light blue polygons in Figure 2). The speculative surfaces are those that either; (a) have a parallel orientation to the local bedding, typically in the Neogene sediments; (b) are not part of a staircase sequence; (c) are dipping slightly landwards rather than seawards; (d) have a geometry/location that could indicate they are river terraces; (e) might have been affected by agricultural structures; or (f) have a slope higher than the SCM threshold, but geometry, position, relative slope and imagery suggest that they are indeed marine terraces.

**Figure 3:**
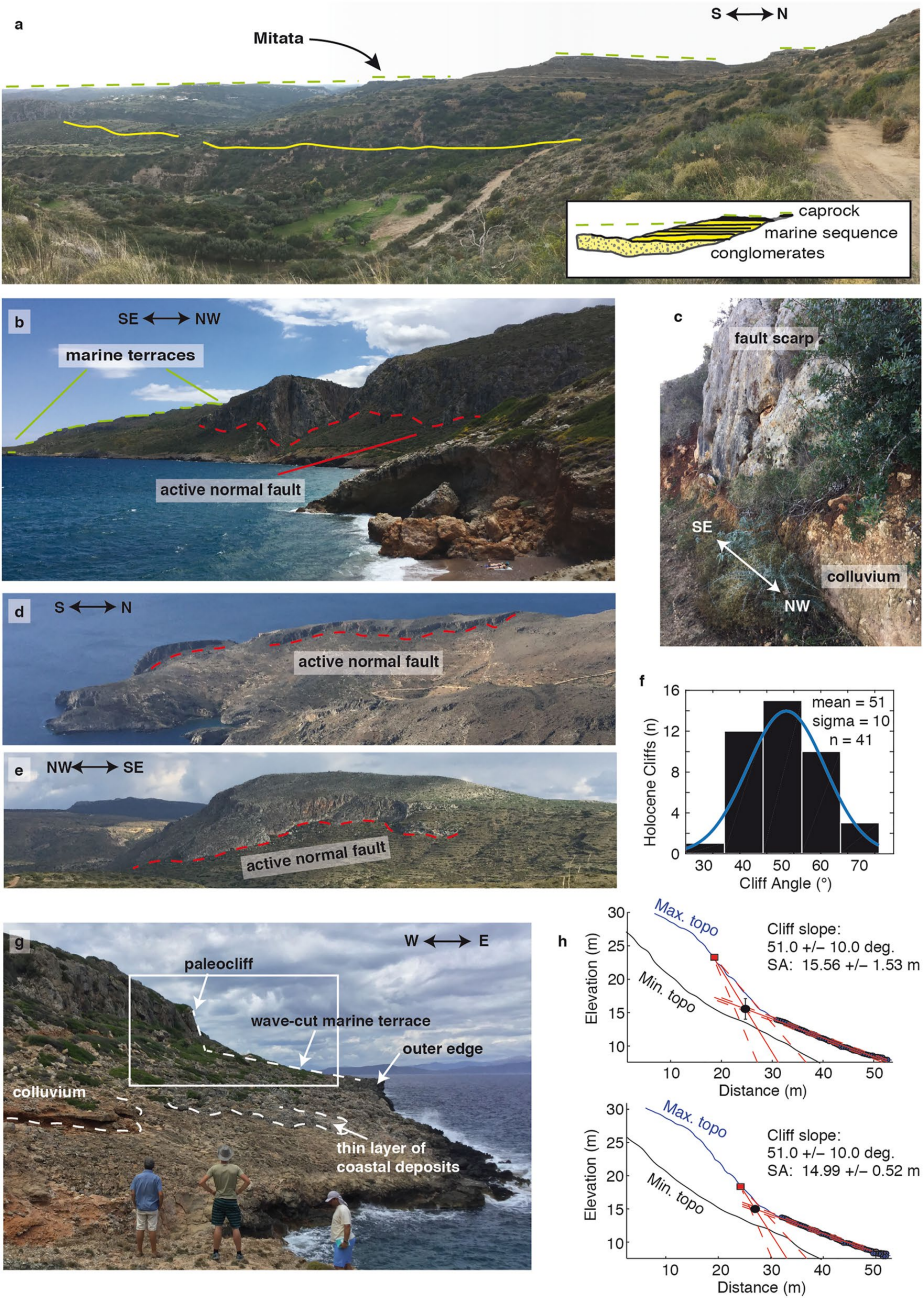
Field photographs and shoreline angle analysis. Locations are given in Figure 2. (a) Section of the basin stratigraphy taken E of Mitata; dashed line outlines the topmost rasa, solid yellow line denotes the top of the conglomerates and the base of the marine sequence; (b) View of terraces and the fault south of Agia Pelagia along the NE‐coast; (c) Detail of active fault scarp near Agia Pelagia; (d) Example of active N‐S striking normal fault near Livadi; (e) Example of active NW‐SE striking fault W of Avlemonas (f) Holocene cliff measurements along NE‐coast; (g) Detail of marine terrace along the NE‐coast near Plateia Ammos; (h) Example of shoreline angle analysis estimating a maximum (top) and minimum (bottom) position of the shoreline angle (SA).

The shoreline angle of marine terraces, at the intersection of paleo‐cliffs and paleo‐platforms, approximates the sea level during former highstands (Lajoie, 1986; Merritts & Bull, 1989), but colluvial wedges caused by cliff degradation often obscure this angle (Hanks et al., 1984). For precise measurements of shoreline angles, we used TerraceM (Jara‐Muñoz et al., 2016), a Matlab® graphical user interface specifically developed to analyse marine terraces from digital topography. Measurements were restricted to the eastern coast, where staircase sequences of terraces are least disturbed by cross‐cutting faults and display a complete section between the coast and the upper rasa (Figure 2). Within the ∼10 km of coastline that we analysed in detail, terraces did not seem to be vertically offset more than ∼10–20 m by faults. From field observations and DSM measurements, we estimated the slope of the present‐day sea‐cliff (51 ± 10°; Figure 3f) and used it as a proxy for the slope of eroded, older sea‐cliffs (Figure 3h). We obtained a range of possible shoreline angle elevations for every terrace in the selected area, by picking a most seaward/landward position of the paleo sea‐cliff on the maximum elevations of cliff‐perpendicular swath profiles and two points of the paleo‐platform (Figure 3; see de Gelder et al., 2015). We made a lateral correlation of terrace levels by comparing elevations of shoreline angles and their morphological characteristics (e.g., platform width, cliff height), using both the map view and stacked swaths (see 3.1).

## 4 Results

### 4.1 Active Faults

**Figure 4:**
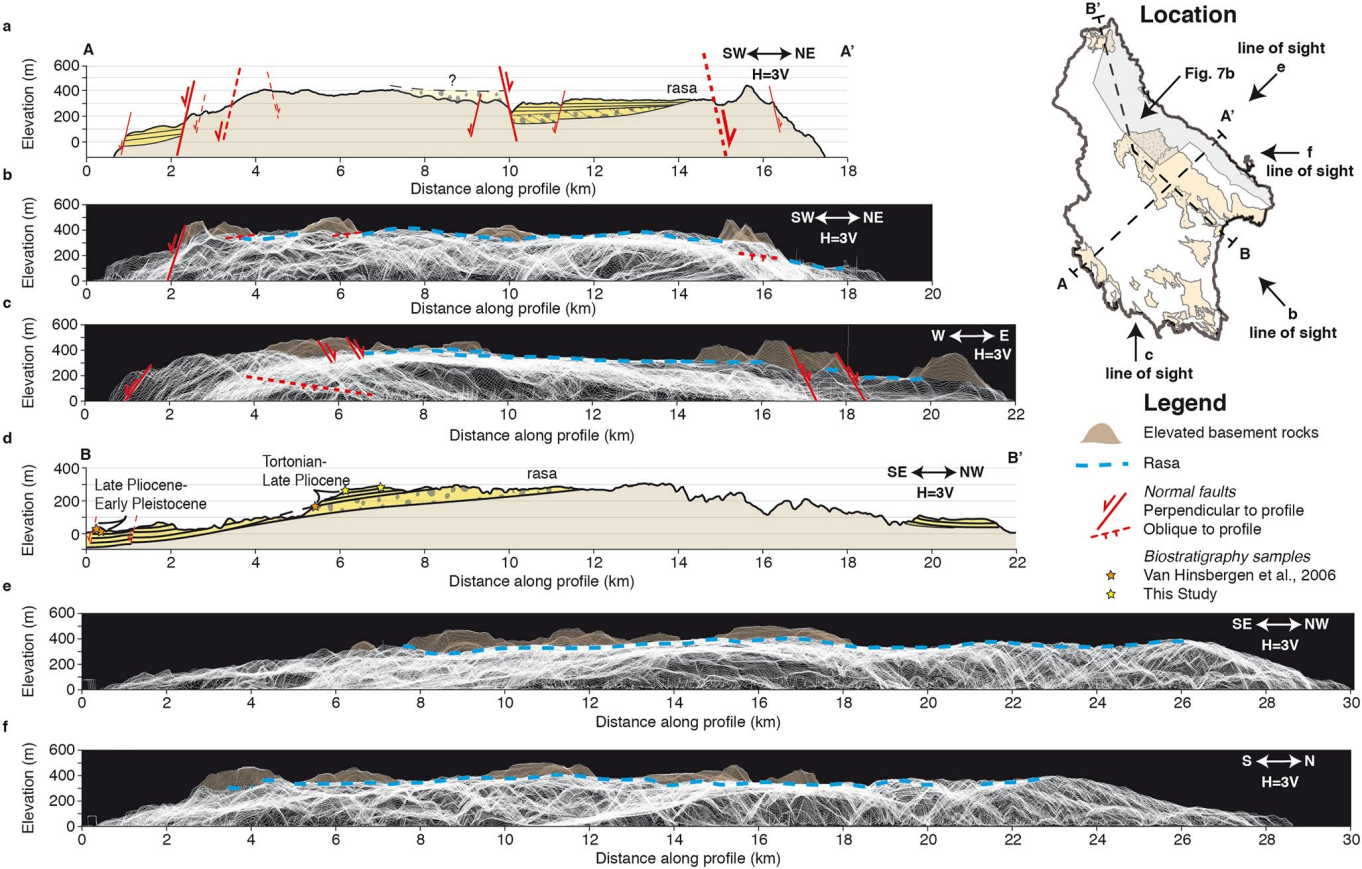
Cross‐sections and island‐scale stacked swath profiles. Top‐right map shows locations of profiles in A‐A’ and B‐B’ on Kythira, as well as the viewing directions of the full‐island stacked swaths in panels b, c, e and f, and the stacked swath location of Figure 7b (a) NE‐SW Cross‐section perpendicular to major basin bounding faults. (b) Stacked swaths along a NE‐SW strike. (c) Stacked swaths along an E‐W strike. (d) NW‐SE Cross‐section along‐strike of the major basin bounding faults. (e) Stacked swaths along a NW‐SE strike (f) stacked swaths along a N‐S strike.

The geometry of most Neogene‐Quaternary sedimentary basins is controlled by NW‐SE trending normal faults. The largest basin, between Potamos and Avlemonas (Figure 2; hereafter Potamos‐Avlemonas Basin), is a half‐graben bounded by faults along its SW margin (Figure 4a), similar to the smaller basin near the coast east of Karvounades. The basins in the south of the island, bounding the SW coast and around Kalamos, Livadi and Kapsali, are partly onlapping on the basement, and partly bounded by NW‐SE trending, and to a lesser extent N‐S trending, normal faults. The basin in the north of the island, W of Plateia Ammos, is mostly onlapping on the basement, apart from its eastern margin, which is bounded by a N‐S trending normal fault dipping to the west. N‐S trending faults are also cross‐cutting the Potamos‐Avlemonas Basin, and the basement at several locations on the island. The area north of Potamos is largely devoid of fault scarps, which are probably unpreserved in the metamorphic rocks of the Arna Unit. Well‐preserved Holocene fault scarps on both N‐S and NW‐SE trending faults indicate that both fault sets are active (examples in Figures 3b–3e). Although we did not carry out systematic measurements, we corroborate the findings of Veliz‐Borel et al. (2022) that fault scarps generally evidence dip‐slip motion. Compared to the fault mapping of Veliz‐Borel et al. (2022) the main difference is that their Group A faults, large NW‐SE striking faults crosscutting the whole island, are much lengthier than we mapped them (Figure S3 in Supporting Information S1). The sections with postglacial fault scarps are similar for both maps, but we did not find clear evidence for active faulting in between those sections, despite our 2 m‐resolution DSM and fieldwork. We note that in their fault length/displacement analysis the Group A faults of Veliz‐Borel et al. (2022) generally have low displacements compared to their fault length, which would be consistent with our interpretation that the Group A fault sections with fault scarps are not connected. From the example cross‐section comparing our fault mapping with that of Veliz‐Borel et al. (2022; Figure S3 in Supporting Information S1), we think our fault mapping better correlates the expected topographic offsets to their causal active normal faults.

**Figure 5:**
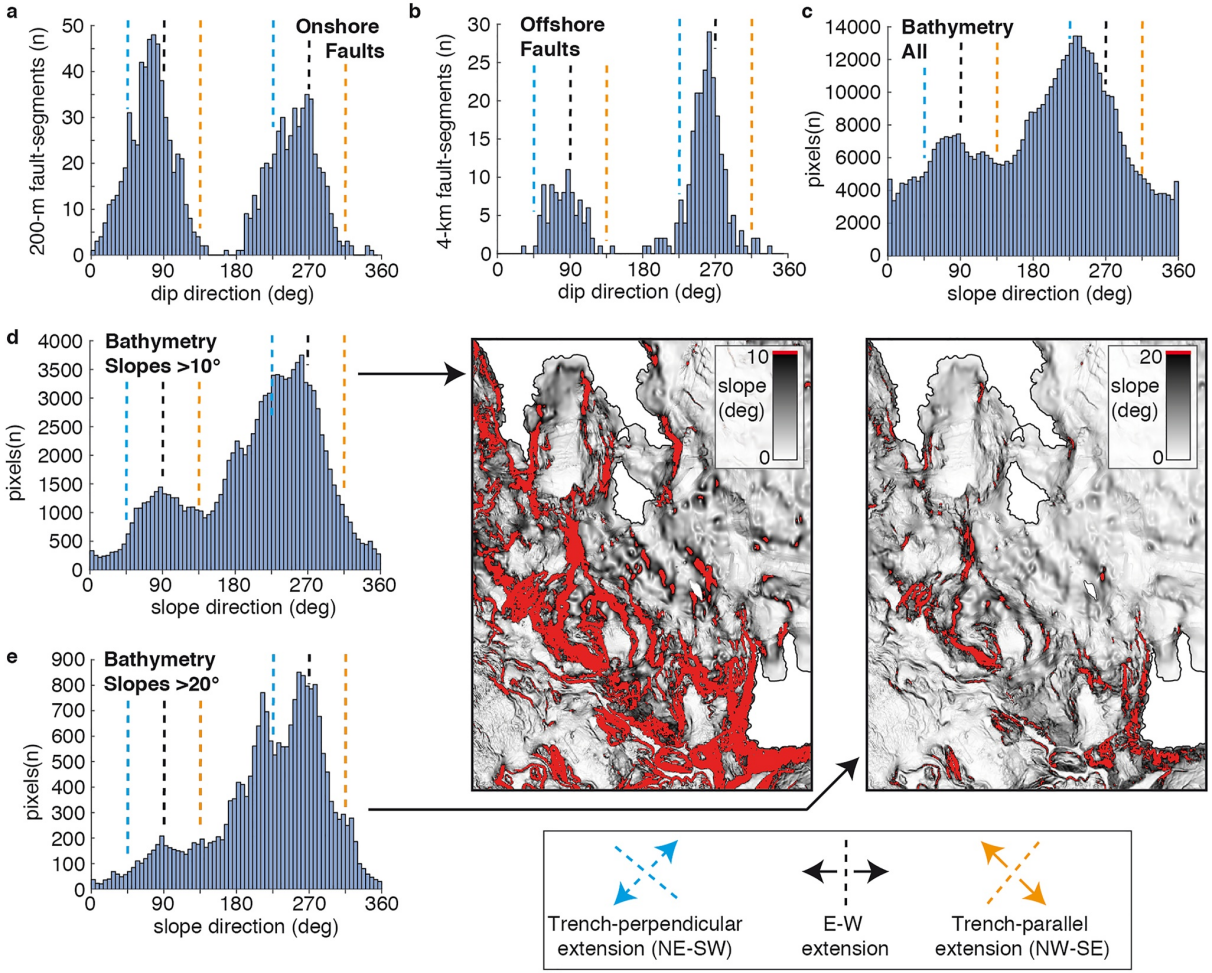
Fault dip directions and slope directions. (a) Fault dip directions of 200‐m fault segments mapped on Kythira in Figure 2. Fault dip directions should be perpendicular to the strike of active normal faults (b) Fault dip directions of 4‐km normal fault segments mapped within the bathymetry of the SW‐Hellenic Arc of Figure 1 (c) Slope direction for all bathymetric data points NE of the Hellenic Trench in Figure 2 (EMODNet bathymetry data). To a first order, the slope direction of steep slopes should be perpendicular to the strike of active faults. (d) Same as (c), but filtering data points that slope more than 10°, as plotted in map‐view below. (e) Same as (d), but for 20°.

The separation of our mapped faults into distinct fault segments 200 m in length (Figure 5a) indicates that N‐S striking faults are dominant on the island, especially E‐dipping ones. NW‐SE trending faults and trends in between N‐S and NW‐SE are also common, although slightly less important. This result is very similar to the faults we mapped offshore in the SW Hellenic Arc (Figure 5b), which trend N‐S predominantly and NW‐SE to a lesser extent. We carried out the same analysis using alternative offshore fault mapping (Figure S4 in Supporting Information S1). The mapping of Veliz‐Borel et al. (2022) is very similar to ours, although they interpret fewer N‐S striking lineaments as faults, resulting in a more equal importance of NW‐SE and N‐S striking faults. In some cases, like the faults SE of Kythira, we mapped the actual segmentation of those faults, which are sets of N‐S trending fault segments in N‐S‐to‐NW‐SE en‐echelon systems, which explains part of the discrepancy. The fault mapping of the National Observatory of Athens (NOA) is generally more variable in terms of strike, but similar to ours, suggests N‐S striking faults are dominant. Taking the overall slope direction of the bathymetry, SW sloping directions toward the Hellenic Trench are the most common (Figure 5c). When filtering out the slope directions for pixels sloping more than 10° or 20°, which should be more representative of active faults, slope directions (dips) to the E and W are dominant (Figures 5d and 5e), suggesting that ∼N‐S striking faults are more active.

These findings collectively indicate that extension is mostly E‐W directed, and thus oblique to the trench direction, both on Kythira and on the scale of the SW Hellenic Arc. Trench‐perpendicular NE‐SW extension has played a dominant role in the formation of Neogene‐Quaternary basins on Kythira bounded by NW‐SE faults (Figures 2 and 5). These faults are still or again active, but appear less important than N‐S striking faults in accommodating active deformation. Trench‐parallel NW‐SE extension (with NE‐SW striking faults) is minor or absent, both on the scale of Kythira and the scale of the SW Hellenic Arc.

### 4.2 Sedimentary Basins

The stratigraphically lowest sedimentary infill in central Kythira consists of continental clastic deposits of variable grain size, including conglomerates, sandstones, siltstones and clays, and reaching a maximum thickness of ∼100 m SE of Potamos (Figures 2, 4, and 4d). The conglomerates that dominate the base of this section become gradually sparser up‐section. These conglomerates are clast supported, and contain sub‐rounded, polymict, and poorly sorted clasts of the three basement units, locally carving the basement with channel geometries. Imbrications and dip of infill deposits in those channels indicate transport toward the NE, similar to the present‐day river reaching the coast near Agia Pelagia (Figure 2). East of Mitata, the overlying marine deposits are deposited conformably on top of the continental deposits (Figures 3a and 4a), in a gradual up‐sequence transition from continental to coastal to shallow marine. Along the NE‐margin of the Potamos‐Avlemonas Basin, marine deposits are deposited directly on top of the basement (Figure 4a). The marine deposits around Mitata and the rasa consist of limestones, sandstones and marls and are rich in *Pectinidae*, oysters and other macro‐fossils, indicating a shallow marine environment. The marine deposits close to Avlemonas city consist mostly of laminated and homogeneous marls intercalated with coarse sandy limestone beds, and are poor in macro‐fossils, possibly indicating a deeper marine environment.

The marine deposits are generally dipping ∼0–10° to the SE (Figure 4), and combined with the geometry of the continental‐marine transition, indicate that the Potamos‐Avlemonas basin is consistently 100–200 m thick, with larger content of continental deposits in its NW margin than toward its SE margin, where marine deposits dominate (Figure 4d).

### 4.3 Micropaleontological Analyses and Biostratigraphy

**Figure 6:**
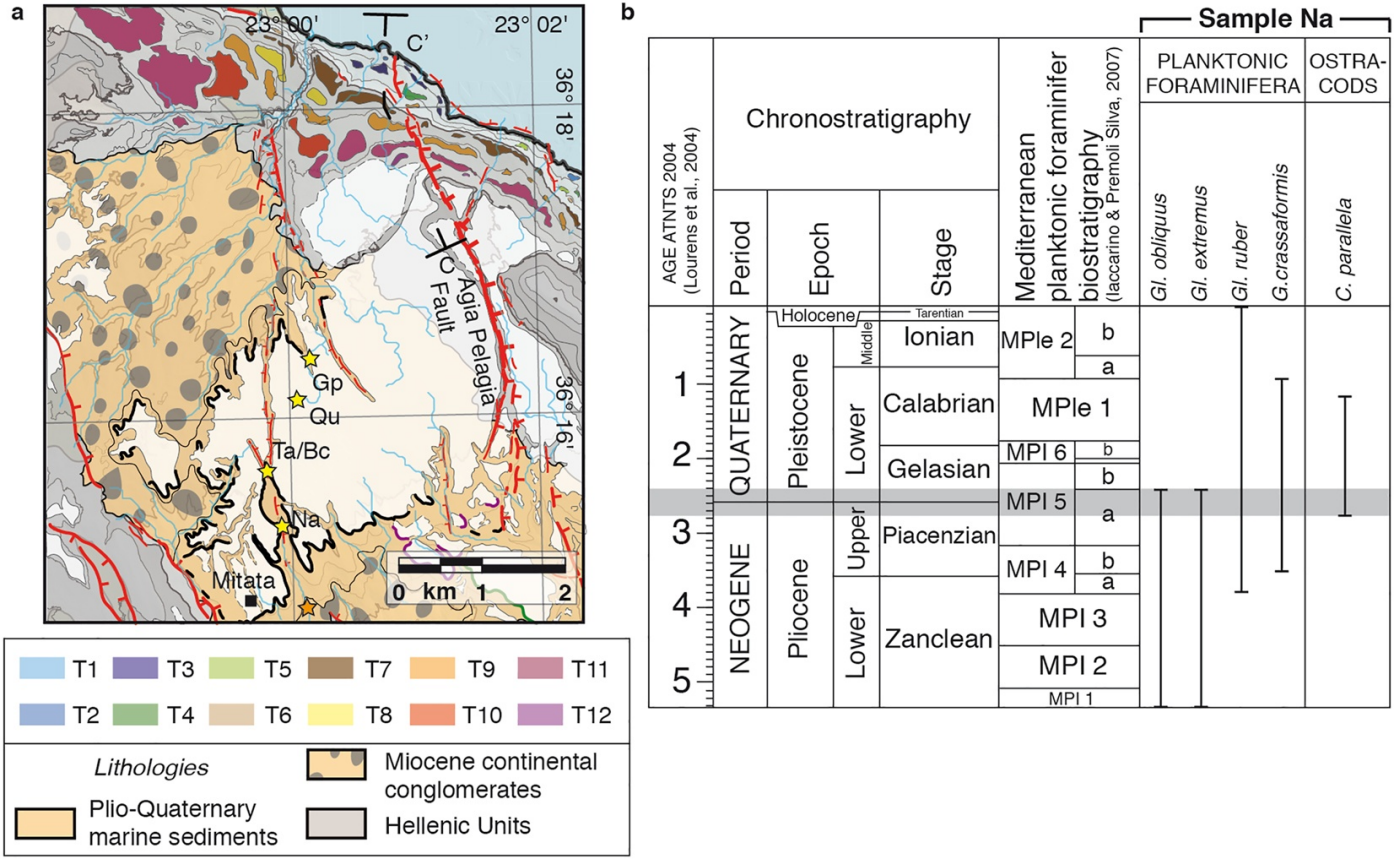
Biostratigraphic dating results. (a) Zoom‐in of Figure 2 with location of samples used for biostratigraphic dating, as well as map of marine terraces for which shoreline angles were determined (Figure 7a), and the location of profile C‐C’ (Figure 8c) (b) Mediterranean planktic foraminifer biostratigraphic scheme of Pliocene‐Quaternary, with the distribution of selected species of planktic foraminifers and ostracods recovered in sample Na. The gray band corresponds to the constrained age of the deposits immediately below the rasa. Timescale is after the Astronomical Tuned Neogene Time Scale 2004 (Lourens et al., 2004), and biostratigraphy after Lirer et al. (2019).

Planktic and benthic foraminifers and ostracods were analyzed from five sites (Figure 6a; Table S1 in Supporting Information S1). Scanning electron microscope images of selected foraminifers and ostracods are presented in Figure S2 of Supporting Information S1. Ostracods were recovered in all the collected samples, while benthic foraminifers were absent in sample Qu. In general, they were well preserved and abundant, albeit not taxonomically diverse. Benthic foraminiferal assemblages consisted of a few reworked (Miocene) species, some eurybathic species and some other species typical of the infralittoral environment (approximately 0–50 m depth), such as *Elphidium* spp. (*E. advenum*, *E. complanatum*, *E. crispum*, *E. macellum*), *Asterigerinata planorbis*, *Aubignyana perlucida*, *Lobatula lobatula*, and *Cancris oblongus* (Di Bella, 2010; Morkhoven et al., 1986; Sgarrella & Moncharmont Zei, 1993). Ostracod assemblages, mainly consisting of *Aurila* spp., *Callistocythere* spp., and *Semicytherura* spp., confirm a shallow infralittoral environment with vegetated bottom. Conversely, planktic foraminifers were collected only in sample Na, where several reworked species were identified together with some long‐ranging (Miocene‐Recent) species, and a few markers, such as *Globorotalia crassaformis*, *Globigerinoides extremus*, *Globigerinoides obliquus* and *Globigerinoides ruber*. On the base of their stratigraphic distribution (Lirer et al., 2019), the co‐occurrence of these species in sample Na and the presence of the ostracod *Callistocythere parallela* in samples NA, Bc and Ta (D’Arpa & Ruggieri, 2004) constrain the age of the deposits immediately below the coastal rasa to the late Piacenzian‐early Gelasian (2.8–2.4 Ma; Figure 6b). Given its elevation of ∼325 m above present sea level, this gives an average long‐term uplift rate of ∼0.11–0.14 mm/yr (same as suggested by Gaki‐Papanastassiou et al., 2011) if sea‐level elevations were comparable to today or up to ∼30 m higher (Dutton et al., 2015). This value is averaged over the last ∼2.6 Ma, and should be taken as a minimum value.

### 4.4 Marine Terraces and Rasa

The wide top‐most rasa covers a large part of the island (Figures 2 and 4), but is not preserved above the metamorphic Arna Unit in the northern part of the island. The very few topographic highs elevated above the rasa would have been paleo‐islands when sea‐level was around rasa elevations, unless footwall uplift elevated them after rasa formation. These highs roughly follow the NW‐SE trend of the basement nappes (Figures 2 and 4). As the rasa is only a few meters above the dated shallow marine deposits, it must be of younger age, ≤2.8–2.4 Ma (see above).

Both the rasa and the marine terraces are preserved within the Pindos and Tripolis basement units as well as in the sedimentary basins, but not within the metamorphic Arna Unit. Apart from the rasa caprock mentioned in Section 3.3, similar caprocks of coastal deposits appear at the highest terrace levels (∼200–300 m elevation) within the Potamos‐Avlemonas Basin (Figure 2), in a low‐angle unconformity with underlying marine basin deposits. Along the NE coast a thin layer of coastal deposits overlying the wave‐cut platform exists at some locations (e.g., Figure 3g), well cemented and containing sparse shell fragments. We assume that these deposits are coeval to paleoshoreline formation, and note that the layer typically dips seaward <5°, which is commonly much less than the bedding of the deformed basement.

**Figure 7:**
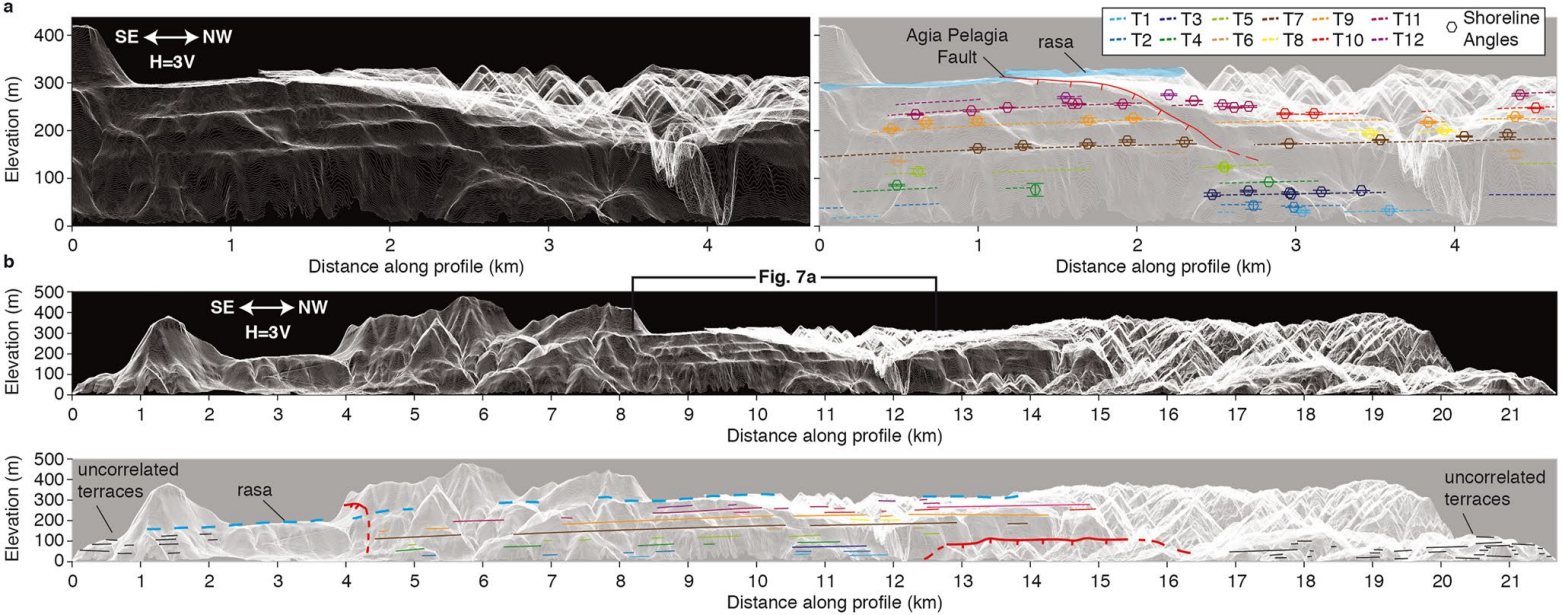
Terraces along the NE coast. (a) Detail of stacked swath profile showing area for which shoreline angles were determined. Thin white lines represent 300 parallel swath profiles of 10 m width, with arrows indicating orientation of profiles. Shoreline angles and error bars of the twelve terrace levels (T1 to T12) are derived similarly to the example given in Figure 3c, with details provided in Figure S6 of Supporting Information S1. The location of the Agia Pelagia Fault is indicated in Figure 6a. (b) Stacked swath profile of whole NE‐coastline (top plot; location in Figure 4), including trends of marine terraces and rasa (bottom plot).

Our detailed analysis of shoreline angles (Figure S6 in Supporting Information S1) reveals that the terraces along the NE coast are slightly tilted along the strike of the coastline over a distance of ∼10 km (Figure 7). The best‐preserved and most continuous terraces are T7, T9 and T11 (Figures 6a and 7), and decrease in elevation by ∼25% over this distance. Other terraces are more scattered but can be correlated laterally following a similar trend. The continuity of marine terraces between the footwall and hanging wall of the Agia Pelagia Fault (Figure 7a) indicates that the fault did not offset the terraces by more than ∼10–20 m.

The *apparent* dip of the coastline toward the SE (Figure 7) may imply a *true* dip approximately S‐, SE‐ or E‐wards. In the case of a simple tilt, stacked swath profiles would show a continuous deformation pattern of paleoshorelines (straight lines) when looking at a perpendicular angle to the tilt (as in De Gelder et al., 2022). Looking at the scale of the whole island along four different projections the rasa is continuously tilted eastward within the northwards view (Figure 4c), varying from ∼400 m elevation in the west to ∼200 m in the east over a distance of ∼15 km, and at a relatively constant ∼400 m elevation in the westwards view (Figure 4f). In the northwestward view the rasa appears lightly, but discontinuously tilted toward the southeast (Figure 4b), and in the southwestward view the rasa appears lightly, but discontinuously tilted toward the northeast (Figure 4e). The 4 viewing directions above support an overall island uplift with an approximately E‐ward tilt. This is best compatible with a N‐S striking fault to the west of Kythira as dominant source of uplift, with other active faults on‐ and offshore contributing less to the first order deformation pattern.

## 5 Discussion

### 5.1 Terrace Correlation

**Figure 8:**
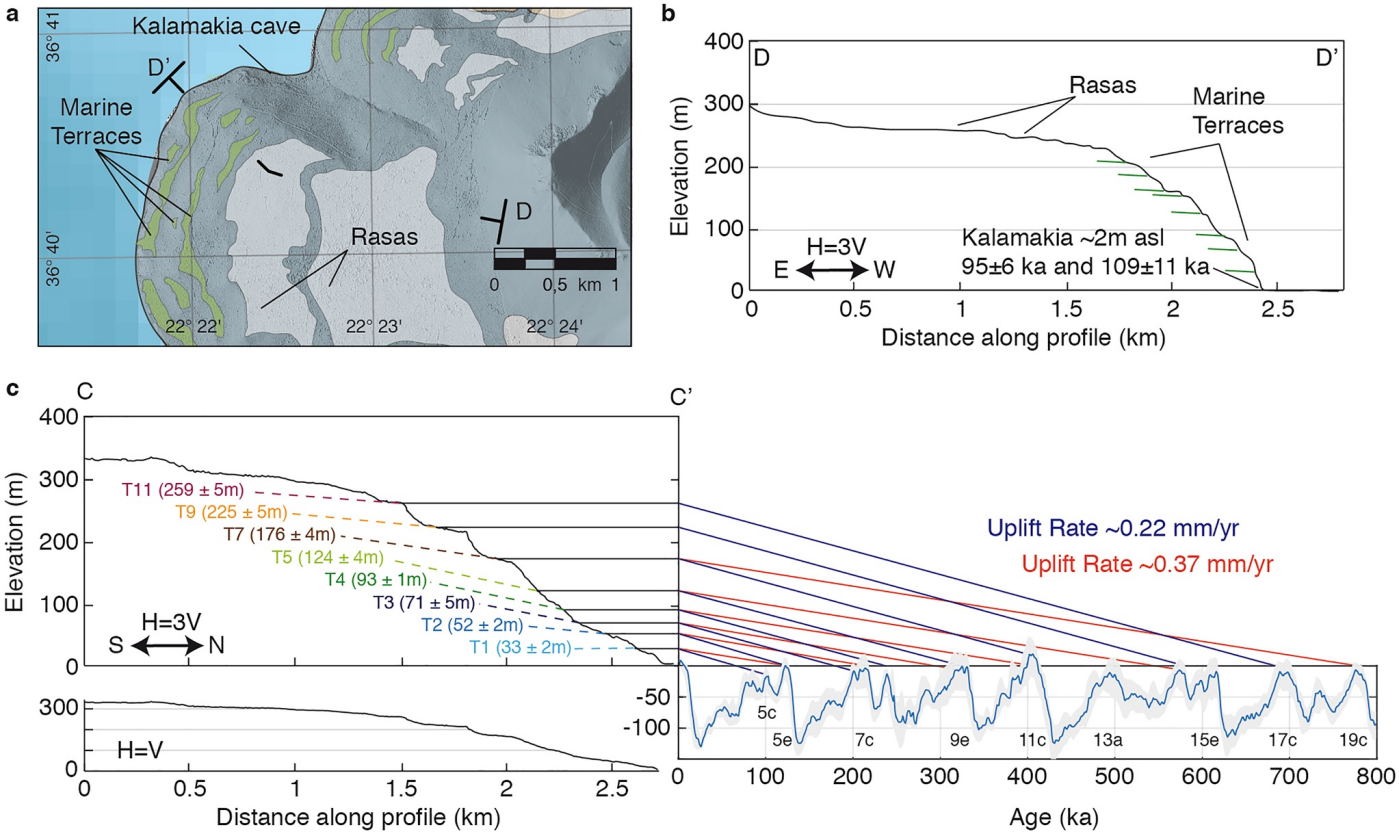
Proposed correlation of marine terraces to sea‐level highstands. (a) Map of terraces/rasas around the Kalamakia cave on the Mani Peninsula (location in Figure 1) (b) Topographic profile D‐D’ of Kalamakia terrace sequence, derived from a Pleiades‐DSM (as used for Kythira) with radiometric ages. (c) Correlation of main terrace levels to sea‐level highstands, using the eustatic sea‐level curve of Spratt and Lisiecki (2016) and the graphical method of Bloom and Yonekura (1990) adopting the shoreline angle elevations of profiles 31, 42 (T1), 18, 42 (T2), 15, 17, 19, 29, 30 (T3), 19 (T4), 16, 17 (T5), 14, 28 (T7), 10, 11 (T9), 11 and 22 (T11) (see Figure S6 in Supporting Information S1). The location of profile C‐C’ is specified in Figure 6a.

Marine terraces that are preserved in uplifting coastlines are typically formed during sea‐level highstands (e.g., Anderson et al., 1999; Lajoie, 1986). The most commonly recorded highstand within marine terrace sequences worldwide is the interglacial Marine Isotope Stage (MIS) 5e (Kopp et al., 2009; Pedoja et al., 2011, 2014). The MIS 5e peaked around 124 ± 5 ka (e.g., Masson‐Delmotte et al., 2010; Stirling et al., 1998) with eustatic sea‐levels around 6 ± 4 m higher than today (Murray‐Wallace & Woodroffe, 2014). Given this, we expect one of the terraces of the sequence on Kythira to have been formed during MIS 5e. However, reliable ages of marine terraces on Kythira are lacking, making terrace attribution difficult. We thus estimate possible correlations of marine terraces to highstands based on the similar morphostratigraphy in the S‐Peloponnese (Figure 8).

Sequences of marine terraces on the Mani and Vatika peninsulas on the S‐Peloponnese (Figure 1) are also capped by wide Plio‐Pleistocene rasas (e.g., Dufaure, 1977; Kelletat et al., 1976; Kleman et al., 2016) at comparable elevations (250–300 m asl) to the rasa in Kythira Island. The only radiometric dates in the region were taken at the Kalamakia cave on the Mani Peninsula (Figure 8a). There, marine deposits ∼2 m above present sea level are tentatively dated with U‐Th as MIS 5c (∼100 ka) (de Lumley et al., 1994), given two samples with ages of 95 ± 6 ka and 109 ± 11 ka. Assuming that this age is accurate, as well as constant uplift rates and a eustatic sea‐level during MIS 5c of 5–40 m below present sea level (Figure 8c; Spratt & Lisiecki, 2016), MIS 5e should be at an elevation of 8–54 m above present sea level. On the Vatika Peninsula, biostratigraphic evidence for Tyrrhenian deposits (∼80–130 ka) is found on marine terraces at different elevations up to ∼20 m above the present‐day sea‐level (Dufaure, 1970; Kelletat et al., 1976; Kowalczyk et al., 1992). The highest and oldest of those deposits (named Euthyrenian; Dufaure, 1970) would correspond to MIS 5e, now at ∼20 m elevation on the Vatika Peninsula. Assuming an uplift rate of the same order of magnitude for Kythira, we estimate the lowest terrace (T1, 31–35 m) or T2 (50–54 m) to correspond to MIS 5e. Taking into account MIS 5e elevation and age uncertainties mentioned above, this would imply uplift rates of ∼0.22 ± 0.04 mm/yr or ∼0.37 ± 0.04 mm/yr, or approximately 0.2–0.4 mm/yr. We extrapolated 0.22 and 0.37 mm/yr uplift rates to correlate marine terraces at higher elevations to older marine sea‐level highstands up to 800 ka (Figure 8c). If uplift rates have indeed been constant between the T1/T2 terraces and the highest well expressed marine terrace (T11), the latter would have an age of ∼1.2–0.7 Ma. If uplift rates have been constant between the T1/T2 terraces and the rasa, the latter would have started emerging from the sea ∼1.5–0.9 Ma.

### 5.2 Uplift History

**Figure 9:**
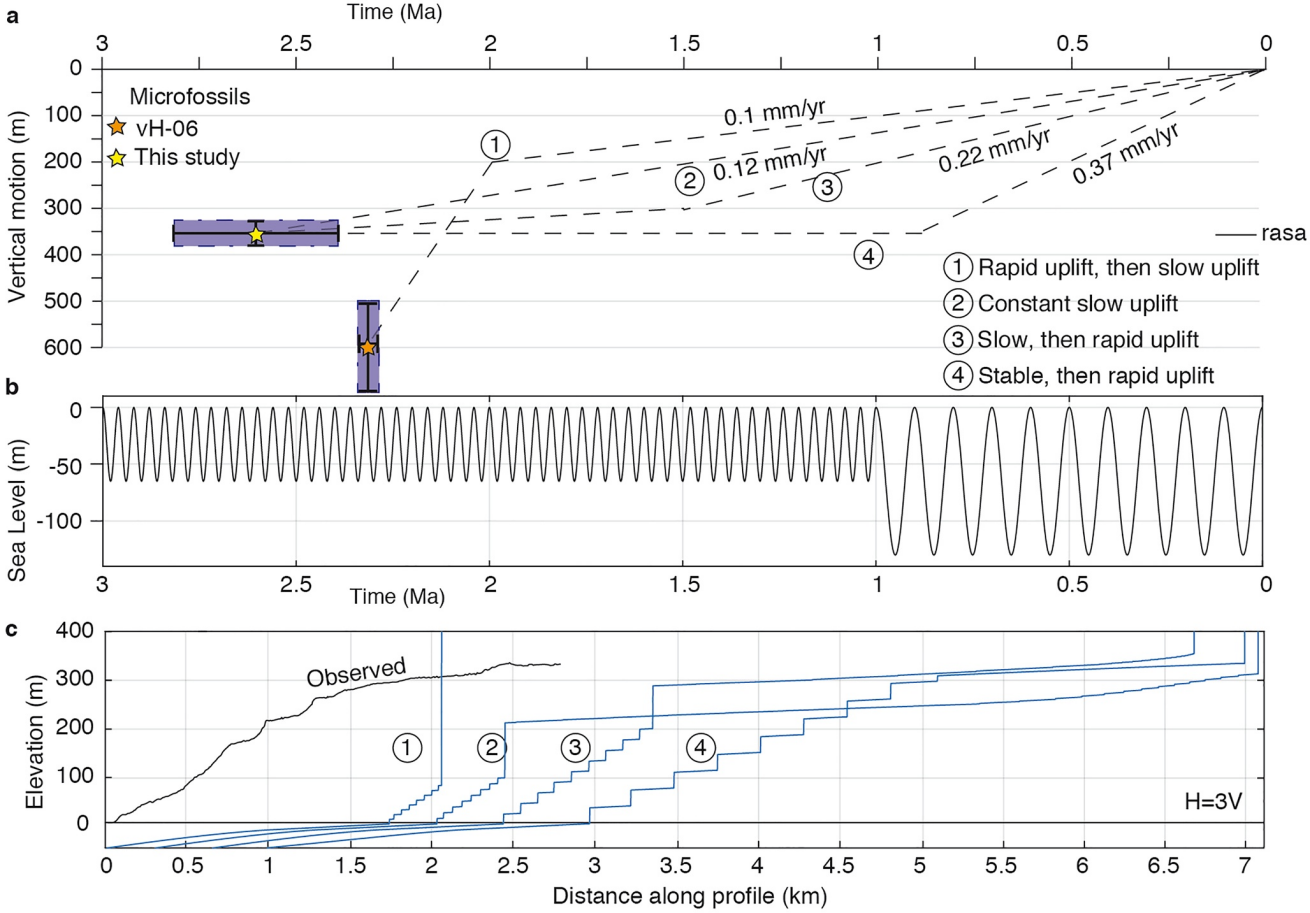
Landscape evolution modeling under different uplift scenarios. (a) Different vertical motion scenarios, with scenario 1 assuming 400 m of rapid uplift followed by 200 m of slow uplift, as suggested by Van Hinsbergen et al. (2006), and scenarios 2, 3 and 4 assuming the dated samples of this study are equivalent to maximum transgression at ∼2.6 Ma, and either steady slow uplift since then (scenario 2), a slow uplift followed by a rapid uplift (scenario 3 ‐ uplift rate 0.22 mm/yr as estimated in Figure 8) or stable conditions followed by a rapid uplift (scenario 4 ‐ uplift rate 0.37 mm/yr as estimated in Figure 8). (b) Extremely simplified sea‐level curve used for the modeling (following de Gelder et al., 2020). (c) Modeling results for the different scenarios, compared to the terraced topography as given in Figure 8. Parameter values are given in Table 2 of Supporting Information S1.

Whereas the age and elevation of the rasa indicate an uplift rate of ∼0.11–0.14 mm/yr averaged over ∼2.6 Ma for the center of the island (Section 4.3), the Late Quaternary uplift rate estimates based on marine terraces are distinctly higher (0.2–0.4 mm/yr). Previous paleobathymetry estimates based on microfossils suggested another uplift history: initial rapid uplift of 2 mm/yr after ∼2.3 Ma, followed by slower uplift of ∼0.1 mm/yr since ∼2 Ma (Van Hinsbergen et al., 2006). To discriminate between these potential scenarios of surface uplift, we used a simple landscape evolution model. We test 4 different scenarios (Figure 9a): (a) initial very rapid uplift (2 mm/yr) followed by slow uplift (0.1 mm/yr); (b) constant slow uplift (0.12 mm/yr); (c) initial slow uplift (0.05 mm/yr) followed by faster uplift (0.22 mm/yr); and (d) stable conditions followed by rapid uplift (0.37 mm/yr). We use the cliff erosion model in TerraceM (Jara‐Muñoz et al., 2019; adapted from Anderson et al., 1999), with standard parameter values for wave height (4 m), erosion rate (0.5 m/yr) and an initial slope based on the approximate slope of the sequence (12°). For simplicity, and to avoid biased results by sea‐level noise inherent to long‐term (>1 Ma) sea‐level curves (de Gelder et al., 2020), we approximate Quaternary sea‐level by combining a 40 ky‐period, 65 m‐amplitude sine function (2.6–1 Ma) and a 100 ky‐period, 130‐m amplitude sine function (Figure 9b). Additional tests with different parameter values and published Plio‐Quaternary sea‐level curves are given in Figure S5 of Supporting Information S1, and do not change the main modeling outcomes.

The resulting first order morphology can be reproduced by the third and fourth scenarios. The first uplift scenario does not match the observed terrace sequence, as the decelerating uplift rate leads to the formation of a high cliff, since increased erosion due to re‐occupation during younger highstands removes the older terraces. Slower uplift rates imply that terrace carving is distributed over a narrower vertical range, a process that was recently illustrated by Malatesta et al. (2021). The second scenario does not match the observed profile either. The change from ∼40 ky period low‐amplitude sea‐level oscillations to ∼100 ky period high‐amplitude sea‐level oscillations around the Mid‐Pleistocene Transition (∼1 Ma; Clark et al., 2006) should lead to a major change in morphology, which is not recorded by the marine terrace sequence in Kythira. In published Plio‐Quaternary sea‐level curves (Bates et al., 2014; Bintanja & V.d. Wal, 2008; de Boer et al., 2010; Rohling et al., 2014) the change in sea‐level oscillations is less abrupt, but also using those sea‐level curves instead of the simplified one, big cliffs are at least twice as high as any cliff observed in the coastal morphology of Kythira (Figure S5 in Supporting Information S1). The third and the fourth scenarios are more compatible with the observed morphology of a continuous marine terrace sequence culminating in a wide rasa around 300 m elevation. Accounting for this, and for the ∼0.22 and ∼0.37 mm/yr uplift rates that are compatible with the marine terrace elevations, we favour an uplift history with initial stable conditions or slow uplift of Kythira from the sea, followed by faster uplift since ∼1.5–0.7 Ma.

### 5.3 Miocene‐Recent Tectonics of Kythira Island

**Figure 10:**
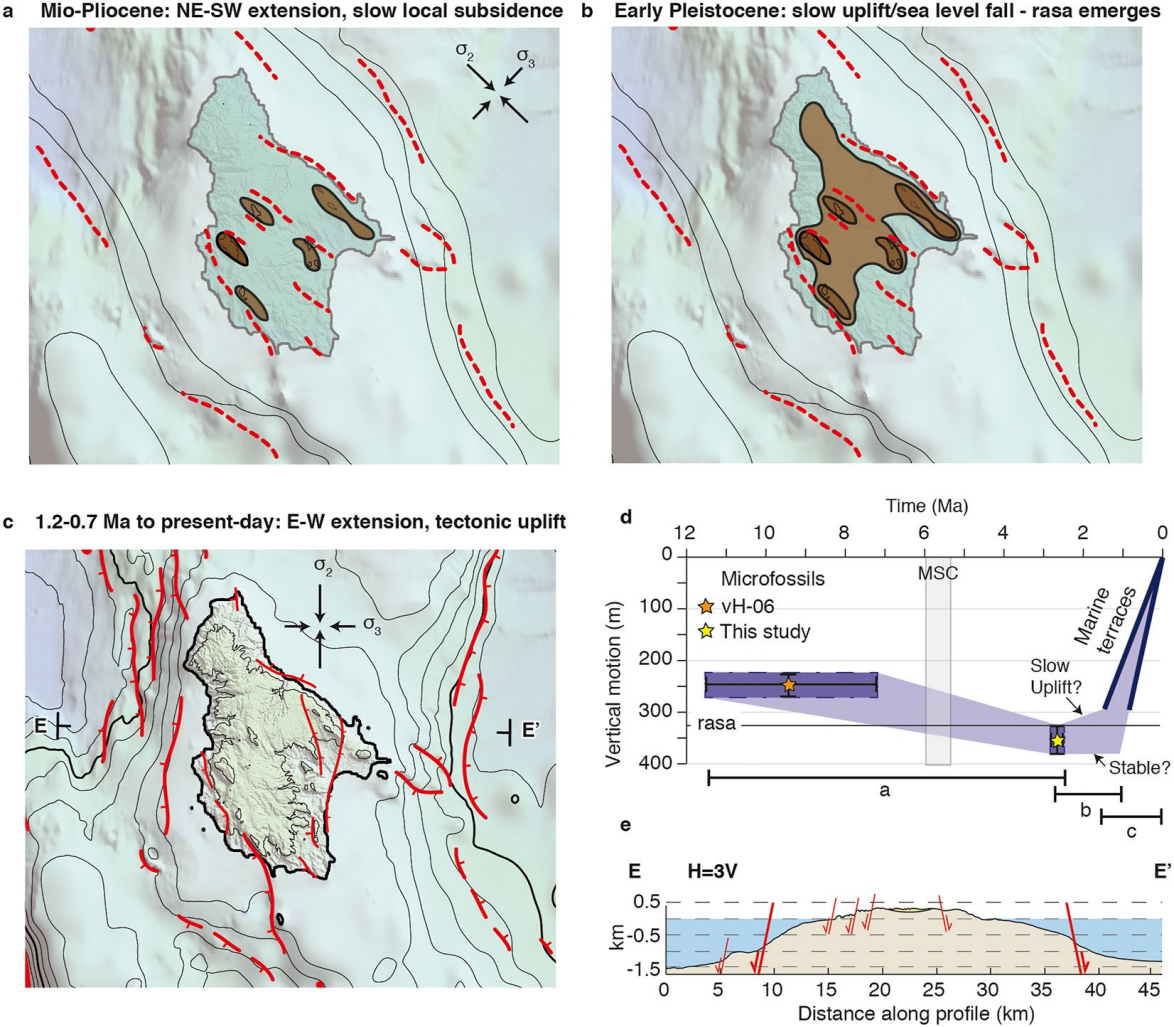
Schematic reconstruction of Miocene‐Recent tectonic history of Kythira. (a) Our interpretation for the phase of NE‐SW extension during the Tortonian‐Pliocene, resulting in NW‐SE oriented topographic ridges and a large, coastal rasa in the latest Pliocene. Dashed lines for fault locations are only illustrative, and not based on data. (b) Our interpretation for the Early Pleistocene, with slow uplift resulting in emergence of the rasa from the sea (c) Present‐day setting, with dominantly E‐W extension and uplift of the island as the results of offshore N‐S trending normal faults. (d) Vertical motion, assuming a paleo‐depth of our samples and the shallow marine Tortonian samples of Van Hinsbergen et al. (2006), of 0–50 m, and a marine terrace uplift rate of 0.22–0.37 mm/yr. (e) Topographic profile E‐E’ across Kythira, see location in (c).

The sedimentary infill in the Potamos‐Avlemonas Basin either infilled a former paleotopography after nappe stacking, or deposited in relation with the NW‐SE striking normal faults that currently bound the basin. We favor the latter, fault‐controlled sedimentation, based on the complete absence of sedimentary deposits within the footwall of the basin‐bounding normal faults (Figure 4). This in turn would support that the ∼100 m of relative subsidence between the Tortonian shallow marine deposits close to Mitata (van Hinsbergen et al., 2006), and the shallow marine Plio‐Pleistocene deposits dated in this study a few kms to the north (Figures 2 and 10), are largely of local origin and due to NE‐SW extension. The geometry, depositional environment and ages of the sediments indicate that the coastline gradually shifted toward the NW with increasing subsidence, while the ∼200 m thick basin transitioned laterally from continental conditions in the NW to marine conditions in the SE (Figure 4). Within this framework, the Plio‐Pleistocene section near Avlemonas (Figure 2) (van Hinsbergen et al., 2006) should have been deposited in a deeper marine environment than our dated deposits immediately below the rasa. The vertical motion trend of van Hinsbergen et al. (2006) is very similar to ours, but when compared to our data, their paleo‐depth estimate suggests much larger vertical motions (∼400 m subsidence, ∼800 m uplift). We did not find clear geologic or geomorphic evidence of marine conditions higher than the topmost rasa, and therefore suspect that the paleo‐depth range of the fossils they considered is biased. We envision that local environmental/hydrodynamic conditions in the studied section of van Hinsbergen et al. (2006) may have led to different ratios of planktonic/benthic foraminifera (%P) with depth compared to their reference %P curve that was derived from the Gulf of Mexico, the Gulf of California, the west coast of the USA and the Adriatic Sea (van der Zwaan et al., 1990).

We did not observe the angular unconformity between continental and marine deposits described by Meulenkamp et al. (1977). As an explanation, we speculate that the Mediterranean sea‐level drop of tens to hundreds of meters during the Messinian Salinity Crisis (MSC; 5.97–5.33 Ma) (Roveri et al., 2014) may have locally resulted in erosive/unconform sedimentary contacts near former river valleys, as described elsewhere in the Mediterranean (Bache et al., 2012).

We interpret the elevations and overall concave morphology of the terraces and overlying rasa as indicative of land motions at pace with sea‐level variations or as a slow apparent uplift followed by faster uplift around ∼1.5–0.7 Ma. The age of the rasa is coeval to that of the Mid‐Pliocene Warm Period (∼3.3–2.9 Ma; Rovere et al., 2014), after which eustatic sea‐levels may have dropped by a few tens of meters (Dutton et al., 2015; Raymo et al., 2011). Therefore, an initial apparent uplift of Kythira could have been an effect of eustatic sea‐level fall, and hence of climatic rather than tectonic origin. The rate and magnitude of the second uplift phase cannot be accounted for by eustatic sea‐level changes alone, and indicates a recent change in tectonic conditions.

### 5.4 Forearc Uplift and Regional Implications

The N‐S trending normal faults cutting through the topmost rasa and the Tortonian‐Pliocene basin, and the associated surface uplift, post‐date ∼2.8–2.4 Ma. This is consistent with the description of Veliz‐Borel et al. (2022) that N‐S striking faults tend to abut against NW‐SE striking faults on Kythira, and are thus younger in age. Although the total displacement on NW‐SE striking faults is possibly larger on Kythira (Veliz‐Borel et al., 2022), our fault analysis, slope direction analysis, focal mechanisms and the overall eastward tilt of the rasa suggest that N‐S striking faults are regionally more important in accommodating active deformation. We suggest that the eastward tilt is due to the ∼5–15 km long N‐S trending normal faults offshore (Figure 10), which resulted in the uplift of Kythira as an asymmetric horst. An eastward tilt is also in agreement with observations by Gaki‐Papanastassiou et al. (2011) that terraces are generally more elevated along the W‐coast compared to the E‐coast. We propose that the offshore N‐S trending faults would thus be of similar age as the N‐S trending faults onshore but with larger vertical throws, >1 km (Figure 10c). Given uplift to subsidence ratios of ∼1:1–2.5 found for normal faults over timescales incorporating the cumulative effects of co‐, post‐ and interseismic deformation (de Gelder et al., 2019; King et al., 1988; McNeill et al., 2005), these N‐S offshore faults can account for the ∼200–400 m of observed uplift. Whereas we find an extreme lower bound for the onset of E‐W extension and uplift of ∼2.8–2.4 Ma, we deem it more likely that these events started as recently as ∼1.5–0.7 Ma.

Our results on Kythira and in the offshore SW Hellenic Arc indicate that active extension is dominantly E‐W oriented at a regional scale, at an oblique angle to the Hellenic Trench and the preceding phase of NE‐SW extension. We also note that also the NW‐SE striking normal faults resulting from that phase are still or again active, and that the pattern of en‐echelon faulting (Figure 1) implies some degree of horizontal shear (Armijo et al., 1992). As shown in both analogue (Henza et al., 2011) and numerical models (e.g., Deng et al., 2018), preceding normal fault systems can be re‐activated by a new phase of extension, even if that new phase has a different extension direction. The overall zigzag‐pattern of the two active fault systems in the W Hellenic Forearc suggests that the NW‐SE striking fault system was moderately well‐developed (Henza et al., 2011) when the new phase of E‐W extension initiated.

Several authors have interpreted the offshore faults in the SW Hellenic Arc as dominantly trench‐normal and trench‐perpendicular normal faults resulting from trench migration and slab‐rollback (e.g., Angelier et al., 1982; Gallen et al., 2014; van Hinsbergen & Schmidt, 2012). E‐W extension is difficult to reconcile with slab‐rollback as a driving mechanism, as that would mostly lead to NW‐SE and NE‐SW extension in the SW Hellenic Arc, and instead suggests that another mechanism must have induced the present‐day deformation field. Sedimentary underplating has been highlighted by other studies as a potentially important driver of vertical motions around the Hellenic Forearc (Gallen et al., 2014; Menant et al., 2020; Ott et al., 2019). Although this can explain the cyclic pattern of Miocene‐Pliocene subsidence followed by Pleistocene uplift on Kythira, and the (re‐)activation of NW‐SE trending normal faults, there is no obvious explanation to link trench‐perpendicular underplating with trench‐oblique E‐W extension. As such, we do not consider sedimentary underplating as a primary mechanism to explain tectonic changes in the SW Hellenic Arc.

**Figure 11:**
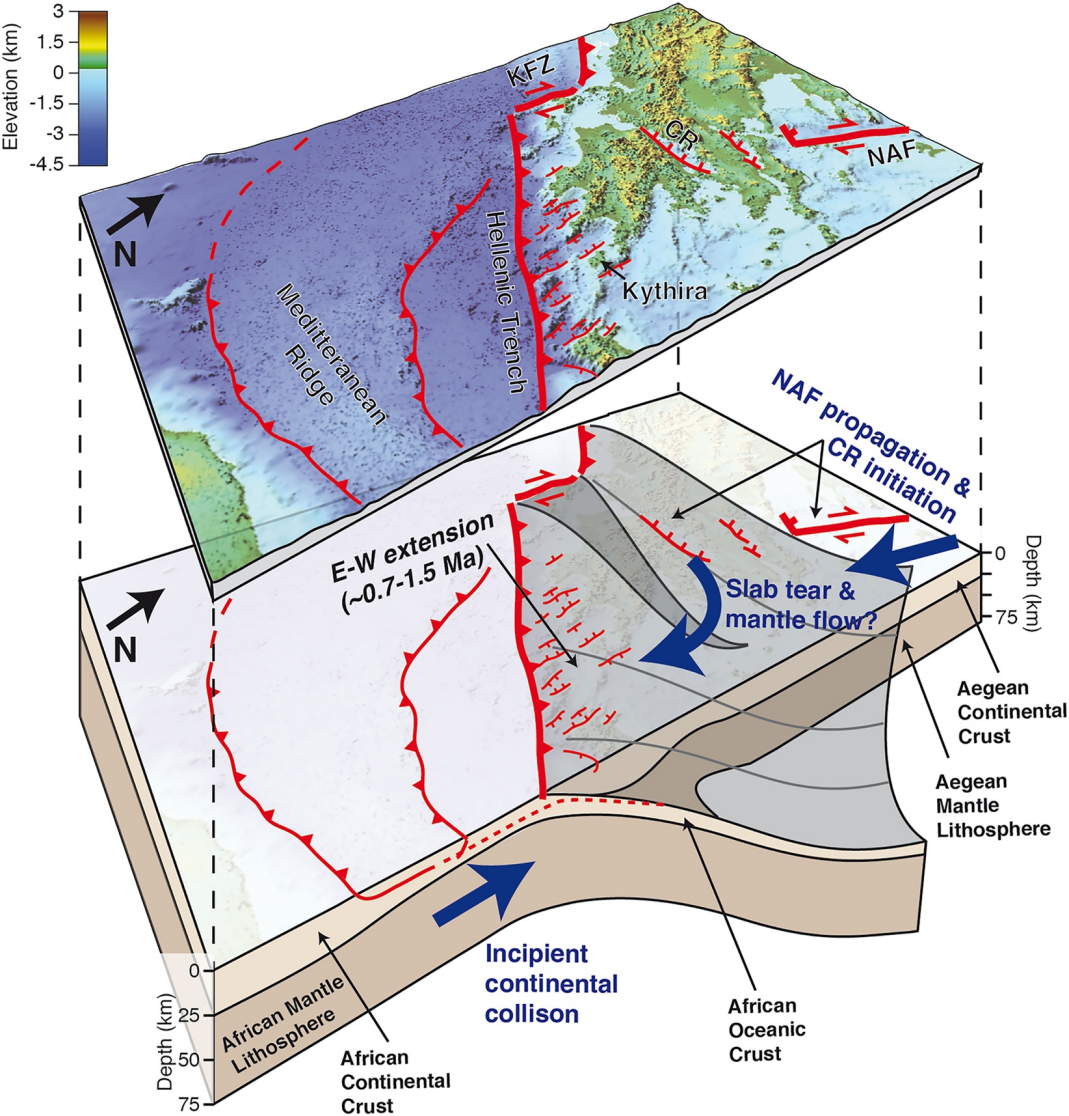
Possible regional drivers of E‐W extension. Top plot shows topo‐bathymetry with main tectonic features, using a Global Mapper (version 15.0) 3D view and a 3x vertical exaggeration. Topography is an ALOS Global Digital Surface Model (DSM), and bathymetry is from the European Marine Observation and Data Network (EMODNet) Digital Terrain Model (DTM). KFZ = Kefalonia Fault Zone, CR = Corinth Rift and NAF = North Anatolian Fault. Bottom plot shows the same area but with underlying African and Aegean lithosphere. Marked in blue are 3 possible mechanisms, not mutually exclusive, that could have led to E‐W extension since ∼1.5–0.7 Ma. This plot is approximately to scale, with African lithospheric structure S of the Hellenic Trench based on Meier et al. (2004), Aegean lithospheric structure based on Jolivet et al. (2013) and schematic geometry of a possible slab tear based on Guillaume et al. (2013). There is some debate on the location of the main plate boundary (e.g., discussion in Shaw & Jackson, 2010), with estimates ranging from the Hellenic Trench to S of the Mediterranean Ridge, but this does not affect our main inferences.

A process that may explain Pleistocene tectonic changes around the forearc is the occurrence of slab tearing, which has been proposed for both the eastern (e.g., Özbakır et al., 2013) and western (e.g., Jolivet et al., 2013; Royden & Papanikolaou, 2011) limits of the Hellenic subduction zone. Given that Kythira is located much closer to the western (∼250 km) than to the eastern limit (∼450 km), especially the W‐limit of the Hellenic Arc is potentially relevant. Analogue models of slab‐tearing, treating the Kefalonia Fault Zone (Figure 11) as the strike‐slip surface expression of the slab tear, suggest toroidal mantle flow would lead to ∼NNE‐SSW directed horizontal lithospheric strain in the Hellenic Forearc (Figure 11; Guillaume et al., 2013). Although this does not perfectly match E‐W extension, strain orientations are close enough that we cannot exclude the possibility that slab tearing plays a role in the SW Hellenic Arc deformation.

As proposed by Lyon‐Caen et al. (1988) and Armijo et al. (1992), the change of extensional regime implies a change in boundary conditions, probably in relation to the nearby subduction zone. They suggested it could be due to incipient collision of the arc with the increasingly buoyant crust of the subducting African margin (Figure 11). A similar tectonic‐scale change in kinematics is supported by a wealth of evidence across the East Mediterranean (e.g., Aksu et al., 2021; Mascle & Chaumillon, 1997; Schattner, 2010), and in agreement with a growing body of observations. Such observations include seismic and tomographic images, local bathymetric mapping and marine basins uplifted on land, all reporting changes in the stress and deformation fields throughout the Mediterranean in the last ∼5 Ma (e.g., Gómez de la Peña et al., 2021; Prada et al., 2020; Zitellini et al., 2020). N‐S trending extensional faults are coherent with the extension direction expected in the overriding plate given collision and N‐S convergence between Anatolia and Africa. The lack of active faults recording E‐W extension in the continent northward possibly reflects the weakness of the Aegean lithosphere there (e.g., Armijo et al., 1992; McKenzie, 1978).

A third possible process to explain tectonic changes in the forearc is the extrusion of the Anatolian plate toward the Aegean domain, associated with the westward propagation of the North Anatolian Fault (NAF; Figure 11; e.g., Armijo et al., 1996, 1999; Flerit et al., 2004). This process has been proposed as the cause of localized extension in the Corinth Rift (Figure 11; e.g., Taymaz et al., 1991; Armijo et al., 1996; Taylor et al., 2011), which is currently extending at plate‐boundary rates of 1–2 cm/yr (Briole et al., 2000). The initiation of localized rapid extension in the Corinth Rift around ∼2–0.6 Ma (Armijo et al., 1996; Fernández‐Blanco et al., 2019a; Gawthorpe et al., 2017; Nixon et al., 2016), is approximately coeval with the initiation of N‐S fault‐driven uplift we propose on Kythira, and we note that river profile inversion on Cret. also suggests accelerated uplift over the past ∼2 Ma (Roberts et al., 2013). The coeval timing of these events support that the recent growth into the Aegean of the propagating NAF may have changed the convergence rate on the Hellenic Trench, possibly doubling it within the Pleistocene (as proposed by Flerit et al., 2004). This could have resulted in stronger mechanical coupling on the deep subduction interface and an increase of the N‐S directed horizontal forces (Flerit et al., 2004), explaining the recent change in extensional fault geometry in the upper crust.

The three processes mentioned above, i.e. incipient collision, NAF propagation and slab tearing, can account for large‐scale tectonic changes in the SW Hellenic Arc individually, but we emphasize that these processes are non‐exclusive. As such, a combination of two or three of these processes, possibly inter‐related, is also a feasible scenario.

In a more general sense, our study of Kythira Island highlights the importance of upper crustal faulting in forearc uplift. As mentioned above, the N‐S striking offshore normal faults explain most, if not all of the observed uplift on Kythira. Deeper seated processes like underplating (e.g., Menant et al., 2020), dynamic topography (e.g., Conrad & Husson, 2009), lower‐crustal mantle flow (e.g., Fernández‐Blanco, 2014; Fernández‐Blanco et al., 2020) and mantle flow in relation to roll‐back and/or slab tearing (e.g., Guillaume et al., 2013) are likely to play a role in any subduction zone around the world, including the SW‐Hellenic Arc, but their relative contributions are difficult to quantify if the forearc is crosscut with upper crustal faults. This is the case further east in the Hellenic Forearc (e.g., Gallen et al., 2014; Howell et al., 2017; Robertson et al., 2019) as well as in many other subduction zones, like the Carribean (e.g., Leclerc et al., 2014), Japan (e.g., Matsu'ura et al., 2009) and New Zealand (e.g., Clark et al., 2010). A key factor to distinguish between uplift sources that we can underline from our study is the deformation pattern and wavelength, specifically perpendicular to the strike of active faults. We know, from well‐resolved examples like the Corinth Rift, that the deformation wavelength of upper crustal normal faults is around ∼15–20 km (de Gelder et al., 2019). Whereas this is compatible with km‐scale tilting on Kythira, the wavelength of lithospheric/mantle scale processes would be much larger, possibly by an order of magnitude (e.g., Buiter et al., 2001; Husson et al., 2022). Continuous deformation markers such as marine terraces and rasas can be especially valuable in that sense, and are thus of primary importance in resolving uplift mechanisms at the front of overthrusting upper plates.

## 6 Conclusions

Our study of the main tectono‐stratigraphic and morphological features of Kythira allows us to draw the following conclusions:

1. Slow local subsidence of ∼100 m within the largest sedimentary basin on Kythira occurred between the Tortonian and Latest Pliocene, possibly largely accommodated by a NW‐SE trending fault system forming a half‐graben.
2. The highest elevated marine deposits below a wide paleo‐coastal rasa constrain maximum transgression to ∼2.8–2.4 Ma, and give a maximum age for the onset of recent uplift.
3. Landscape evolution modeling combined with uplift rate estimates of the marine terrace sequence suggest that the marine regression that followed rasa‐formation occurred in two steps. The first phase occurs with stable conditions or slow uplift, possibly as an effect of eustatic sea‐level drop instead of a tectonic origin. This phase was followed by a faster uplift phase of ∼0.2–0.4 mm/yr that initiated around 1.5–0.7 Ma.

Our evidence supports that this last and ongoing phase of faster uplift is largely driven by N‐S trending normal faults that are part of a regionally dominant mode of active E‐W extension, although also NW‐SE striking normal faults remain active. We attribute the change in tectonic regime and vertical motion around 1.5–0.7 Ma to changing boundary conditions at the subduction zone in relation to incipient collision with the African plate, Aegean‐Anatolian extrusion and propagation of the North Anatolian Fault, and/or slab tearing. In all those cases, increased N‐S directed horizontal forces could have resulted in a new direction of extension and active uplift of horsts like Kythira. In general, we emphasize the importance of upper crustal faulting in dictating forearc uplift patterns.

## Conflict of Interest

The authors declare no conflicts of interest relevant to this study.

## Supporting information

Supporting Information S1

## Data Availability

The Pleiades satellite imagery was obtained through the ISIS and Tosca programs of the CNES under an academic license and is not for open distribution. On request, we'll provide the DSM calculated from this imagery to any academic researcher who gets approval from CNES (contact isis-pleiades@cnes.fr for quoting this paper, and with lacassin@ipgp.fr in copy). We do share a georeferenced hillshade image and slope map of the 2 m‐resolution Digital Surface Model that was developed from Pleiades satellite imagery. This image can be retrieved with these links: https://doi.org/10.6084/m9.figshare.18715535.v1 (hillshade image) https://doi.org/10.6084/m9.figshare.18714914.v1 (slope map). The map of Figure 2 can be downloaded in georeferenced format (as Geospatial PDF) at https://doi.org/10.6084/m9.figshare.18703496.v1. Procedures to reproduce the analyses are described in the text.
