# Supplementary material for: Quaternary E‐W Extension Uplifts Kythira Island and Segments the Hellenic Arc
Source: Tectonics. 2022 Oct 11;41(10):e2022TC007231. doi: 10.1029/2022TC007231 (PMC9828760; doi:10.1029/2022TC007231)
Supplement: Supplementary file 1 — Supporting Information S1 [file TECT-41-0-s001.pdf]

AGU PUBLICATIONS

Tectonics

Supporting Information for

Quaternary E-W extension uplifts Kythira Island and segments the Hellenic Arc

G. de Gelder[1,2], D. Fernández-Blanco[2,3], N. Öğretmen[4,5], S. Liakopoulos[6], D. Papanastassiou[6], C. Faranda[5], R. Armijo[2], and R. Lacassin[2]

[1]ISTerre, Université Grenoble-Alpes, 1381 Rue de la Piscine, 38400 St. Martin d'Hères, France

[2]Université de Paris, Institut de physique du globe de Paris, CNRS, F-75005 Paris, France

[3]Barcelona Center for Subsurface Imaging, Passeig Marítim de Barceloneta 37-49, E-08003 Barcelona, Spain

[4]Eurasia Institute of Earth Sciences, Istanbul Technical University, 34467, Istanbul, Turkey

[5]Dipartimento di Scienze, Università degli Studi Roma Tre, Largo San Leonardo Murialdo, 1, 00146 Rome, Italy

[6]Institute of Geodynamics, National Observatory of Athens, GR-11810 Athens, Greece

Contents of this file

Figures S1 to S6
Tables S1 to S2

Additional Supporting Information (Files uploaded separately)

We share a georeferenced hillshade image and slope map of the 2 m-resolution Digital Surface Model through these links: https://doi.org/10.6084/m9.figshare.18715535.v1 (hillshade image) and https://doi.org/10.6084/m9.figshare.18714914.v1 (slope map). The map of Figure 2 can be downloaded in georeferenced format (as Geospatial PDF) at https://doi.org/10.6084/m9.figshare.18703496.v1.

Introduction

Figure S1 is a comparison between Digital Surface Models produced with ASTER or Pleiades imagery (as in this study). Figure S2 shows some Scanning Electron Microscope images for some of the microfossils used in the biostratigraphic dating section. Figure S3 shows a comparison of our fault mapping on Kythira and the fault mapping of Veliz-Borel et al. (2022). Figure S4 shows a comparison of our offshore fault mapping to those of two other studies. Figure S5 shows sensitivity tests of the landscape evolution modeling. Figure S6 are detailed topographic cross-sections used to determine shoreline angles of marine terraces. Table S1 lists all microfossils found in the samples, and Table S2 the model parameters used for the landscape evolution modeling.

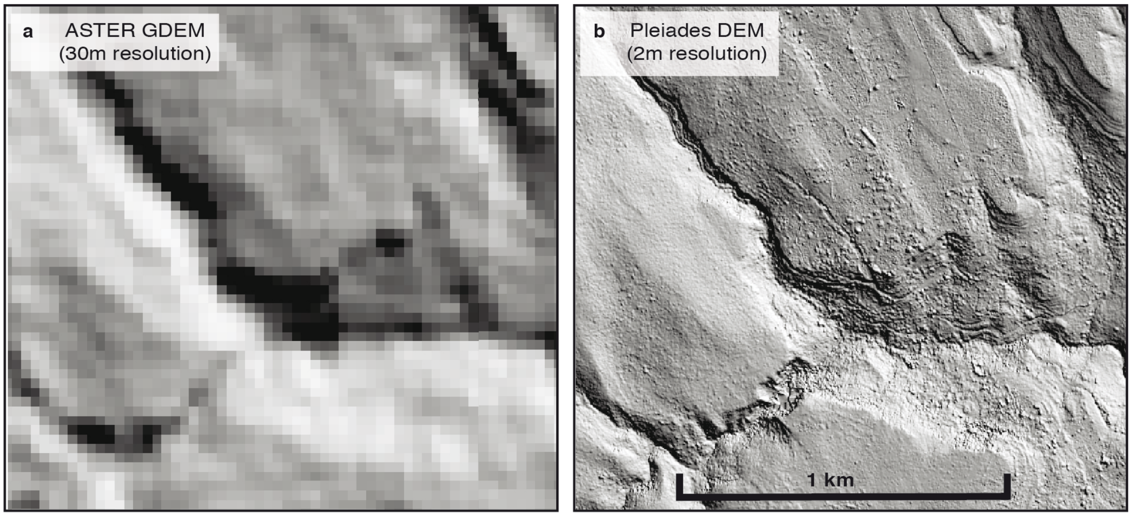

Figure S1. Topographic data comparison. Comparison between the standard Aster GDEM (30m horizontal resolution) and the DSM (2m horizontal resolution) we produced with images of the Pleiades satellite; location given by inset in Fig. 2.

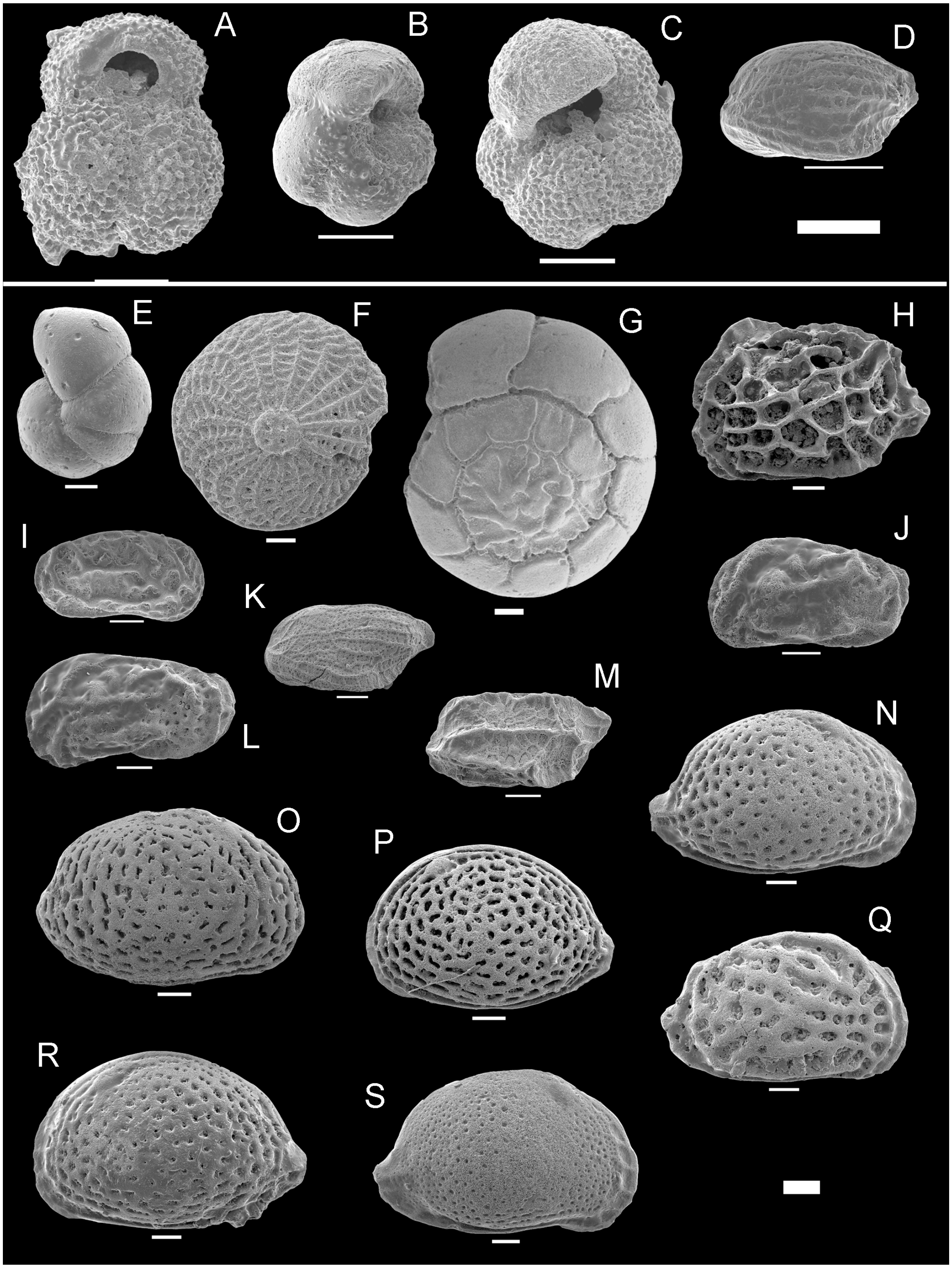

Figure S2. SEM pictures of selected planktic and benthic foraminifers and ostracods collected in the shallow marine deposits in Kythira. A. *Globigerinoides ruber*, sample Na; B.

Globorotalia crassaformis, sample Na; C. *Neogloboquadrina atlantica atlantica*, sample Na; D. *Semicytherura inversa*, sample Na; E. *Lobatula lobatula*, sample Gp; F. *Elphidium crispum*, sample Ta; G. *Aubignyana perlucida*, sample Ta; H. *Mutilus elegantulus*, sample Ta; I. *Callistocythere flavidofusca*, sample Na; J, *Callistocythere parallela*, sample Ta; K., *Semicytherura velata*, sample Ta; L. *Callistocythere intricatoides*, sample Ta; M. *Semicytherura* sp., sample Ta; N. *Aurila punctata*, sample Ta; O. *Aurila cephalonica*, sample Gp; P. *Aurila* sp., sample Ta; Q. *Cimbaurila cimbaeformis*, sample Ta; R. *Aurila anguisfoveata*, sample Ta; S. *Aurila hesperiae*, sample Ta. White bars correspond to 0.1 mm.

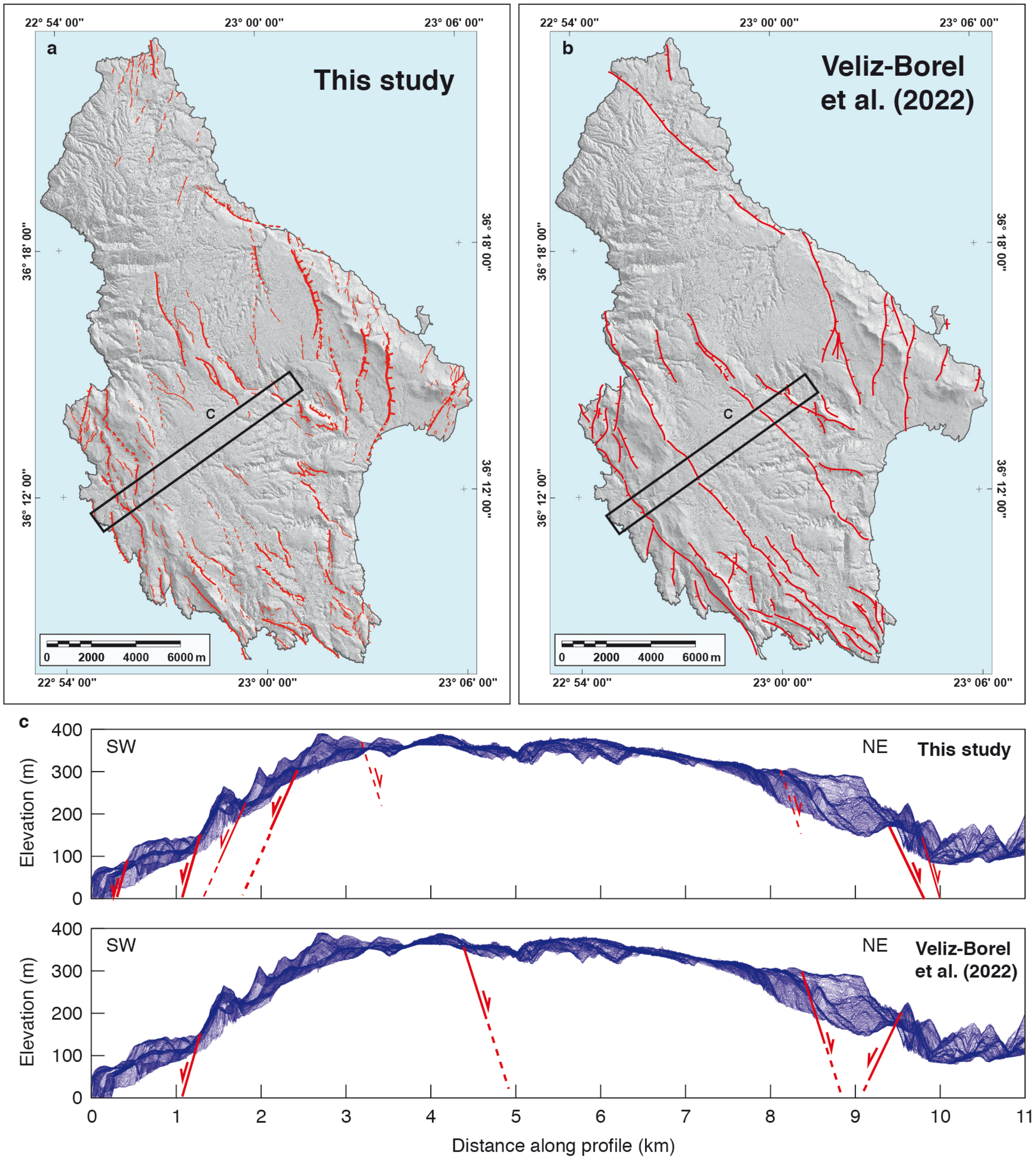

Figure S3. Onshore fault analysis comparison. (a) Kythira Island fault mapping from our study, same as Fig 1. (b) Same area with mapping of Veliz-Borel et al. (2022) (c) Stacked swath profile for the area marked in a and b, with fault locations as mapped in our study (top) and in Veliz-Borel et al. (2022) (bottom). Fault depth and dip are schematic only, and not based on measurements. Vertical exaggeration is ~5 times.

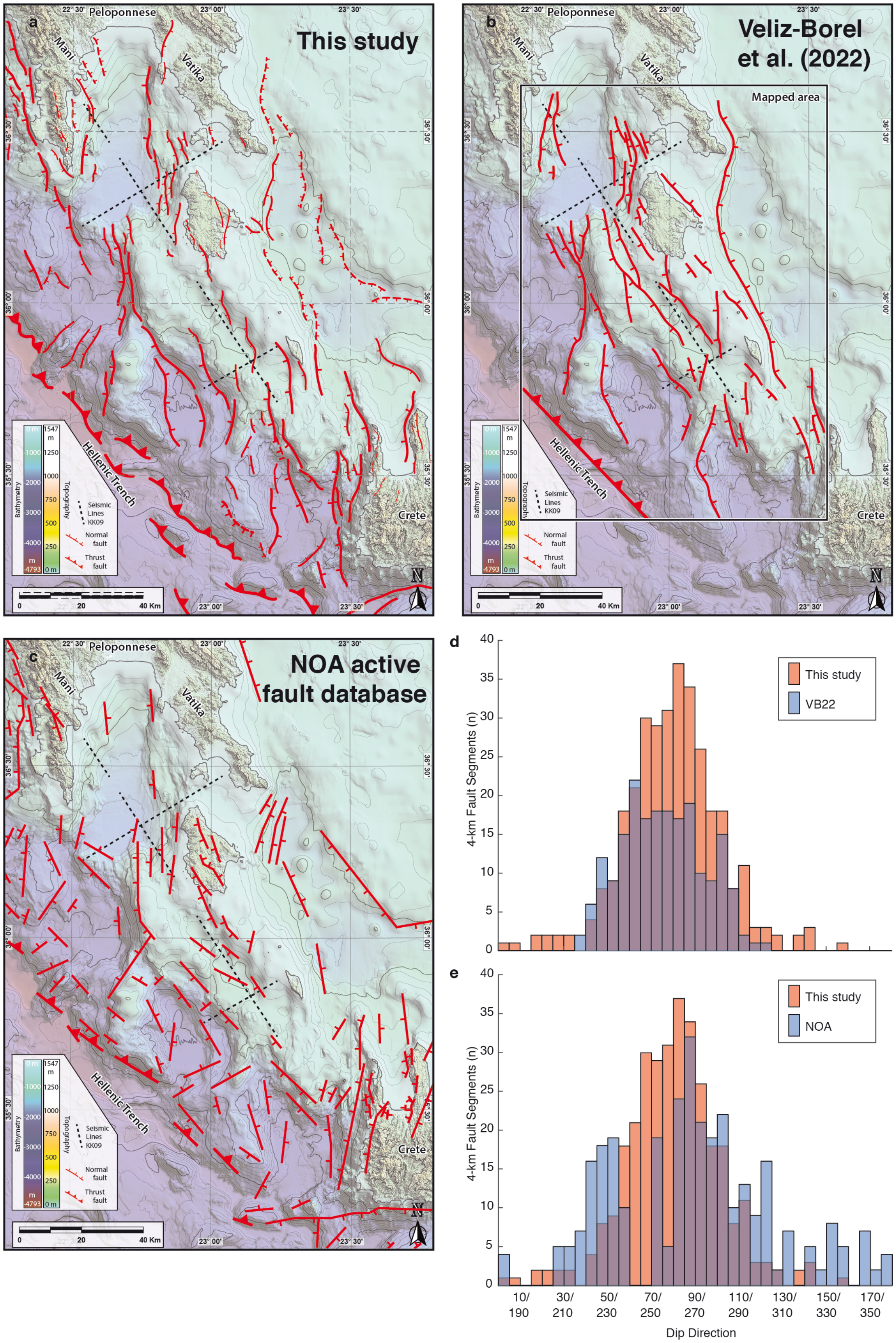
a
This study
b
Veliz-Borel
et al. (2022)
Mapped area
c
NOA active
fault database
Peloponnese
Mani
Vatika
Hellenic Trench
Crete
Bathymetry
Topography
Seismic Lines KK09
Normal fault
Thrust fault
0
20
40 Km
d
This study
VB22
4-km Fault Segments (n)
e
This study
NOA
Dip Direction
10/ 190
30/ 210
50/ 230
70/ 250
90/ 270
110/ 290
130/ 310
150/ 330
170/ 350

Figure S4. Offshore fault analysis comparison. (a) Regional fault mapping of the SW-Hellenic Arc from our study, same as Fig 1. Dashed lines are the seismic lines of Kokinou and Kamberis (2009) (b) Same region with mapping of Veliz-Borel et al. (2022) (c) Same region with faults as they are mapped in the National Observatory of Athens (NOA) active fault database (d) Fault dip directions of 4-km normal fault segments mapped by Veliz-Borel et al. (2022), compared to the dip directions as mapped in this study (e) Same as **d**, but for the NOA database

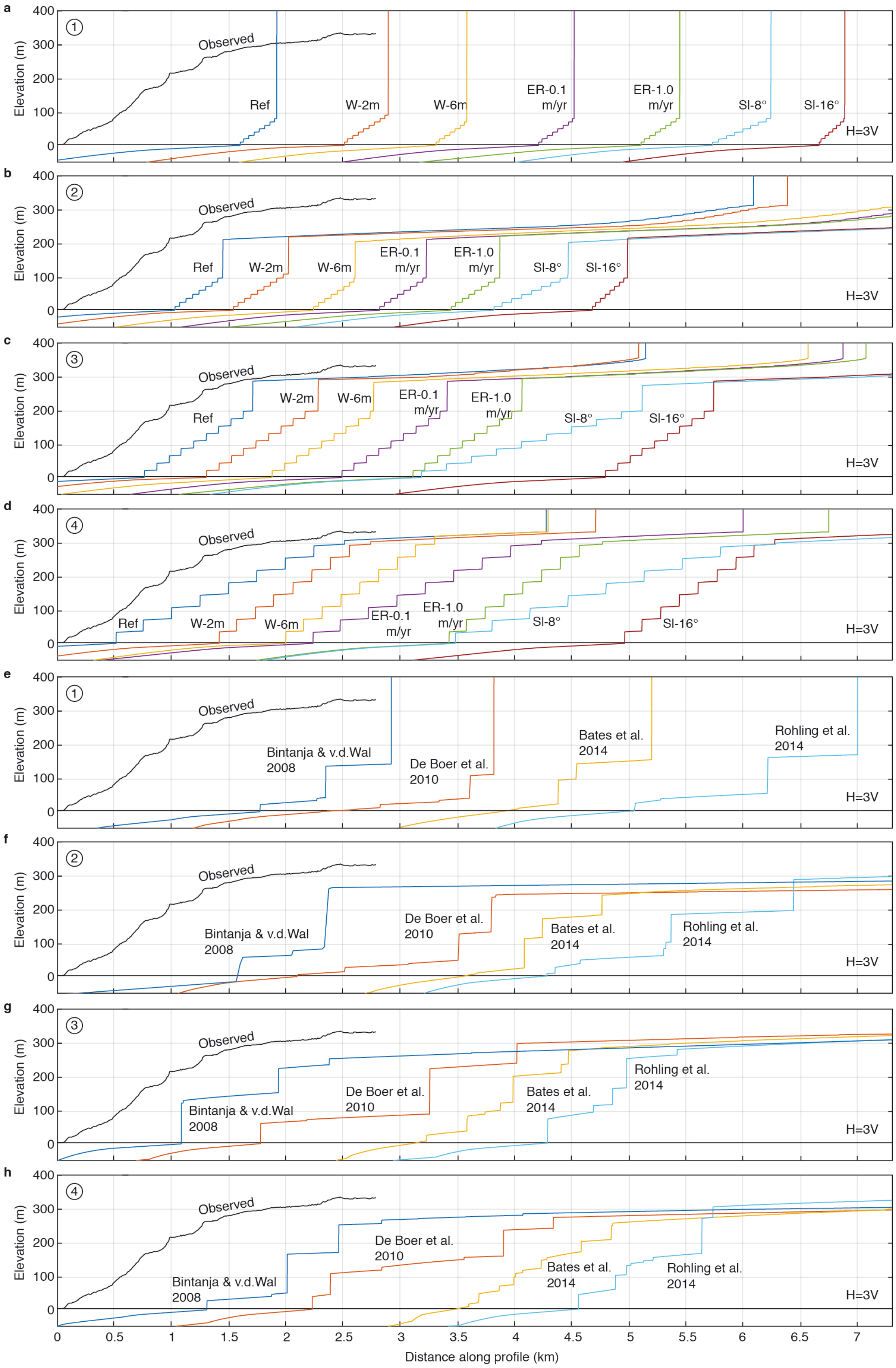
a
b
c
d
e
f
g
h
Elevation (m)
400
300
200
100
0
Observed
Ref
W-2m
W-6m
ER-0.1 m/yr
ER-1.0 m/yr
Sl-8°
Sl-16°
H=3V
Bintanja & v.d.Wal 2008
De Boer et al. 2010
Bates et al. 2014
Rohling et al. 2014
0
0.5
1
1.5
2
2.5
3
3.5
4
4.5
5
5.5
6
6.5
7
Distance along profile (km)

Figure S5. Sensitivity tests for LEM modeling. (a-d) Testing the influence of wave depth, erosion rate and initial slope within reasonable ranges on the 4 tested uplift rate scenarios of Fig. 9. (e-h) Using the Bintanja and van der Wal (2008), De Boer et al. (2010), Bates et al. (2014) and Rohling et al. (2014) sea-level curves (as in De Gelder et al., 2020) on the 4 tested uplift rate scenarios, with the same reference parameter values as in Fig. 9 (also see Supplementary Table 2). As the Bintanja and van der Wal (2008) only spans 3 Ma, values for the 4-3 Ma period have been copied from the De Boer et al. (2010) sea-level curve, which was derived with a similar methodology.

Figure S5 is 15 pages and can be found through:
https://doi.org/10.6084/m9.figshare.18986210.v1

Figure S6. Swath profiles used to determine shoreline angles. Map gives locations and numbering of swath profiles on a slope-map of the Pleiades DSM, and the average determined shoreline angles (dots). Color-code for the marine terraces is the same as in Fig. 8. Profiles show maximum and minimum topography, selected points on the terrace and paleo-cliff, and estimated shoreline angle elevation for two end-member scenarios: a most landward and a most seaward position of the hypothetical paleo sea-clifff.

Sample BC (2.87 – 2.59 Ma)

BC Benthic foraminifera

*Asterigerinata planorbis (*d'Orbigny, 1826)
Astrononion stelligerum (d'Orbigny, 1839)
Cibicides refulgens (de Montfort, 1808)
Cibicidoides pseudungerianus (Cushman, 1922)
Elphidium complanatum (d'Orbigny, 1839)
Elphidium crispum (Linnaeus, 1758)
Elphidium macellum (Fichtel & Moll, 1798)
Lobatula lobatula (Walker & Jacob, 1798)
Melonis soldanii (d'Orbigny, 1846)
*Uvigerina pygmaea (*d'Orbigny, 1826)

BC Ostracods

Aurila anguisfoveata (Uliczny, 1969)
Aurila cephalonica (Mostafawi & Matze-Kasarsz, 2006)
Aurila convexa (Baird, 1850)
Bairdia sp.
Buntonia conularis (Terquem, 1878)
Callistocythere intricatoides (Ruggieri, 1953)
Callistocythere parallela (Aruta, 1986)
Carinocythereis whitei (Baird, 1850)
Costa edwardsii (Roemer, 1838)
Cystacythereis rubra (Müller, 1894)
Cytherella sp.
Cytheretta subradiosa (Roemer, 1838)

Cytheropteron sp.
Echinocythereis pustulata (Namias, 1900)
Eucytherura patercoli (Mistretta, 1967)
Graptocythere intricata (Terquem, 1878)
Hemicytherura gracilicosta (Ruggieri, 1953)
Loxoconcha ovulata (Costa, 1863)

Mutilus elegantulus (Ruggieri & Sylvester-Bradley, 1875)
Paracytheridea sp.
Ruggieria sp.
Semicytherura inversa (Seguenza, 1880)
Semicytherura velata (Ciampo, 1985)
Semicytherura sp. 1
Semicytherura sp. 2
Semicytherura sp. 3
Urocythereis exedata (Uliczny, 1969)
Xestoleberis communis (Müller, 1891)

Sample GP (3.81 – 2.59 Ma)

GP Benthic foraminifera

Asterigerinata planorbis (d'Orbigny, 1826)
Cibicides refulgens (de Montfort, 1808)

GP Ostracods

Aurila anguisfoveata (Uliczny, 1969)
Bairdia sp.
Callistocythere flavidofusca (Ruggieri, 1950)
Carinocythereis whitei (Baird, 1850)
Caudites calceolatus (Costa, 1853)
Celtia quadridentata cephalonica (Uliczny, 1969)
Cystacythereis sp.
Cytheretta subradiosa (Roemer, 1838)
Eucytherura patercoli (Mistretta, 1967)
Hemicytherura gracilicosta (Ruggieri, 1953)
Loxoconcha ovulata (Costa, 1863)
Pontocythere turbida (Müller, 1894)
Ruggieria sp.
Sagmatocythere versicolor (Müller, 1894)
Semicytherura velata (Ciampo, 1985)
Tenedocythere exornata (Terquem, 1878)

Urocythereis exedata (Uliczny, 1969)
Urocythereis sp.

Sample NA (2.87 – 2.42 Ma)

NA Planktonic foraminifera

Globigerina bulloides (d'Orbigny, 1826)
*Globigerina falconensis (*Blow, 1959)
Globigerinella pseudobesa (Salvatorini, 1956)
Globigerinita parkerae (Loeblich & Tappan, 1957)
Globigerinoides elongatus (d'Orbigny, 1839)
Gobigerinoides extremus (Bolli, 1957)
Globigerinoides obliquus (Bolli, 1957)
Globigerinoides ruber (d'Orbigny, 1839)
Globigerinoides trilobus (Reuss, 1850)
Globorotalia crassaformis (Galloway & Wissler, 1927)
*Neogloboquadrina atlantica atlantica (*Berggren, 1972)
Neogloboquadrina sp.

NA Benthic foraminifera

*Amphycorina scalaris (*Batsch, 1791)
Asterigerinata planorbis (d'Orbigny, 1826)
Brizalina catanensis (Seguenza, 1862)
Brizalina dilatata (Reuss, 1850)
Brizalina sp.
Cancris oblongus (Williamson, 1858)
Cibicidoides pseudungerianus (Cushman, 1922)
Elphidium aculeatum (d'Orbigny, 1846)
Elphidium advenum (Cushman, 1922)
Elphidium macellum (Fichtel & Moll, 1798)
Lenticulina orbicularis (d'Orbigny, 1826)
Lobatula lobatula (Walker & Jacob, 1798)

Elphidium crispum (Linnaeus, 1758)
Lobatula lobatula (Walker & Jacob, 1798)

Melonis soldanii (d'Orbigny, 1846)
Planulina ariminensis (d'Orbigny, 1826)
Pleurostomella alternans (Schwager, 1866)
Sphaeroidina bulloides (d'Orbigny, 1826)

*Uvigerina peregrina (*Cushman, 1923)
Uvigerina proboscidea (Schwager, 1866)

NA Ostracods

Aurila hesperiae (Ruggieri, 1975)
Aurila punctata (von Munster, 1830)
Bairdia sp.
Callistocythere flavidofusca (Ruggieri, 1950)
Callistocythere parallela (Aruta, 1986)
Carinocythereis whitei (Baird, 1850)

Costa sp.
Cystacythereis sp.
Cytherella sp.
Echinocythereis pustulata (Namias, 1900)
Eucytherura patercoli (Mistretta, 1967)
Graptocythere intricata (Terquem, 1878)
Hemicytherura gracilicosta (Ruggieri, 1953)
Loxoconcha ovulata (Costa, 1863)
Mutilus elegantulus (Ruggieri & Sylvester-Bradley, 1875)
Parakrithe sp.
Puloniella hellenica (Mostafawi, 1989)
Pontocythere turbida (Müller, 1894)
Ruggieria sp.
Semicytherura inversa (Seguenza, 1880)
Semicytherura velata (Ciampo, 1985)
Semicytherura sp. 1
Tenedocythere sp.
Urocythereis sp.
Xestoleberis communis (Müller, 1891)

Sample QU (3.81 – 2.59 Ma)

QU Ostracods

Aurila cephalonica (Mostafawi & Matze-Kasarsz, 2006)
Aurila convexa (Baird, 1850)
Aurila hesperiae (Ruggieri, 1975)
Bairdia sp.
Callistocythere intricatoides (Ruggieri, 1953)

Callistocythere flavidofusca (Ruggieri, 1950)
Carinocythereis whitei (Baird, 1850)
Cystacythereis rubra (Müller, 1894)
Cytheretta subradiosa (Roemer, 1838)
Hemicytherura gracilicosta (Ruggieri, 1953)
Loxoconcha ovulata (Costa, 1863)

Semicytherura inversa (Seguenza, 1880)
Semicytherura velata (Ciampo, 1985)
Tenedocythere exornata (Terquem, 1878)
Urocythereis exedata (Uliczny, 1969)
Xestoleberis communis Müller, 1891

Sample TA (2.87 – 2.59 Ma)

TA Benthic foraminifera

Aubignyna perlucida (Heron-Allen & Earland, 1913)
Brizalina arta (MacFadyen, 1931)
Cibicidoides pseudungerianus (Cushman, 1922)
Elphidium crispum (Linnaeus, 1758)
Lobatula lobatula (Walker & Jacob, 1798)
Melonis soldanii (d'Orbigny, 1846)
Neoconorbina terquemi (Rzehak, 1888)
Pleurostomella alternans
Uvigerina peregrina Cushman, 1923

TA Ostracods

Aurila anguisfoveata Uliczny, 1969
Aurila hesperiae Ruggieri, 1975
Aurila punctata (von Munster, 1830)
Aurila sp. 1
Bairdia sp.
Buntonia conularis (Terquem, 1878)
Callistocythere intricatoides (Ruggieri, 1953)
Callistocythere parallela Aruta, 1986
Callistocythere sp.
Carinocythereis whitei (Baird, 1850)
Cimbaurila cimbaeformis (Seguenza, 1883)
Cymbaurila venetiensis (Uliczny, 1969)
Costa punctatissima Ruggieri, 1962
Cystacythereis rubra (Müller, 1894)

Cytherella sp.
Echinocythereis pustulata (Namias, 1900)
Loxoconcha ovulata (Costa, 1863)
Mutilus elegantulus Ruggieri & Sylvester-Bradley, 1875
Puloniella hellenica Mostafawi, 1989
Sagmatocythere cf. *S. napoliana* (Puri, 1963)
Semicytherura inversa (Seguenza, 1880)
Semicytherura velata Ciampo, 1985
Semicytherura sp. 1
Semicytherura sp. 2

Mutilus elegantulus (Ruggieri & Sylvester-Bradley, 1875)	*Tenedocythere exornata* (Terquem, 1878)
Paracytheridea sp.	*Urocythereis exedata* Uliczny, 1969
Pontocythere turbida (Müller, 1894)	*Urocythereis* sp.
Sagmatocythere versicolor (Müller, 1894)	*Xestoleberis communis* Müller, 1891

Table S1. Micropaleontology. List of all the identified species in the analyzed samples from Kythira

Parameter	Value			
Dx (m)	2			
Dt (yr)	50			
Wave Height (m)	4			
Erosion Rate (m/yr)	0.5			
Slope (degree)	12			
Scenario 1 Age (ka)	2300	2005	2000	0
Scenario 1 UR (mm/yr)	2.0	2.0	0.1	0.1
Scenario 2 Age (ka)	4000	2605	2600	0
Scenario 2 UR (mm/yr)	-0.1	0	0.12	0.12
Scenario 3 Age (ka)	4000	1505	1500	0
Scenario 3 UR (mm/yr)	-0.05	0.05	0.22	0.22
Scenario 4 Age (ka)	4000	905	900	0
Scenario 4 UR (mm/yr)	0	0	0.37	0.37

Table S2. Model parameters. Values used for the landscape evolution modeling analysis presented in Fig. 9 and described in section 5.2. Landscape evolution modeling was done using TerraceM-2 (Jara-Muñoz et al., 2019). Sensitivity tests with wave heights of 2 and 6 m, erosion rates of 0.1 and 1.0 m/yr, and slopes of 6° and 18° are given in Supplementary Figure 3
